# Designer drugs: mechanism of action and adverse effects

**DOI:** 10.1007/s00204-020-02693-7

**Published:** 2020-04-06

**Authors:** Dino Luethi, Matthias E. Liechti

**Affiliations:** 1 Center for Physiology and Pharmacology, Institute of Pharmacology, Medical University of Vienna, Währinger Strasse 13a, 1090 Vienna, Austria; 2 Institute of Applied Physics, Vienna University of Technology, Getreidemarkt 9, 1060 Vienna, Austria; 3 Division of Clinical Pharmacology and Toxicology, University Hospital Basel and University of Basel, Schanzenstrasse 55, 4056 Basel, Switzerland

**Keywords:** Designer drug, New psychoactive substance, Stimulant, Synthetic opioid, Synthetic cannabinoid, Psychedelic

## Abstract

Psychoactive substances with chemical structures or pharmacological profiles that are similar to traditional drugs of abuse continue to emerge on the recreational drug market. Internet vendors may at least temporarily sell these so-called designer drugs without adhering to legal statutes or facing legal consequences. Overall, the mechanism of action and adverse effects of designer drugs are similar to traditional drugs of abuse. Stimulants, such as amphetamines and cathinones, primarily interact with monoamine transporters and mostly induce sympathomimetic adverse effects. Agonism at μ-opioid receptors and γ-aminobutyric acid-A (GABA_A_) or GABA_B_ receptors mediates the pharmacological effects of sedatives, which may induce cardiorespiratory depression. Dissociative designer drugs primarily act as *N*-methyl-d-aspartate receptor antagonists and pose similar health risks as the medically approved dissociative anesthetic ketamine. The cannabinoid type 1 (CB_1_) receptor is thought to drive the psychoactive effects of synthetic cannabinoids, which are associated with a less desirable effect profile and more severe adverse effects compared with cannabis. Serotonergic 5-hydroxytryptamine-2A (5-HT_2A_) receptors mediate alterations of perception and cognition that are induced by serotonergic psychedelics. Because of their novelty, designer drugs may remain undetected by routine drug screening, thus hampering evaluations of adverse effects. Intoxication reports suggest that several designer drugs are used concurrently, posing a high risk for severe adverse effects and even death.

**Electronic supplementary material:**

The online version of this article (10.1007/s00204-020-02693-7) contains supplementary material, which is available to authorized users.

## Introduction

The term “designer drugs” was originally introduced to describe novel substances that are derived from clandestine alterations of well-known drugs of abuse, preserving or enhancing pharmacologic effects while remaining outside of legal control (Jerrard 1990). The term is currently applied more widely to include substances that originate from industrial or academic research but never receive medical approval. Some substances that are referred to as designer drugs may be medically approved in different countries, thus not fitting the classic definition of a designer drug (Bäckberg et al. 2019; Manchester et al. 2018; Owen et al. 2016; Zawilska and Wojcieszak 2019). The Internet plays a crucial role in the distribution of designer drugs and in the acquisition of information about them (Miliano et al. 2018). The number of available designer drugs is constantly growing, and trends and patterns of use change over time. This poses a challenge to drug-regulatory authorities and can jeopardize public health. Designer drugs can generally be divided into the same categories as traditional drugs of abuse, namely stimulants, sedatives, dissociatives, cannabinoids, and psychedelics. However, in contrast to traditional drugs of abuse, newly emerging drugs can remain undetected by routine drug screening, and information about associated adverse effects is often scarce. Knowledge of the mechanism of action and potential clinical complications of designer drugs is key for healthcare workers who treat intoxicated patients. The present review provides an overview of the main mechanisms of action and adverse effects of currently available designer drugs.

## Stimulants

**Fig. 1:**
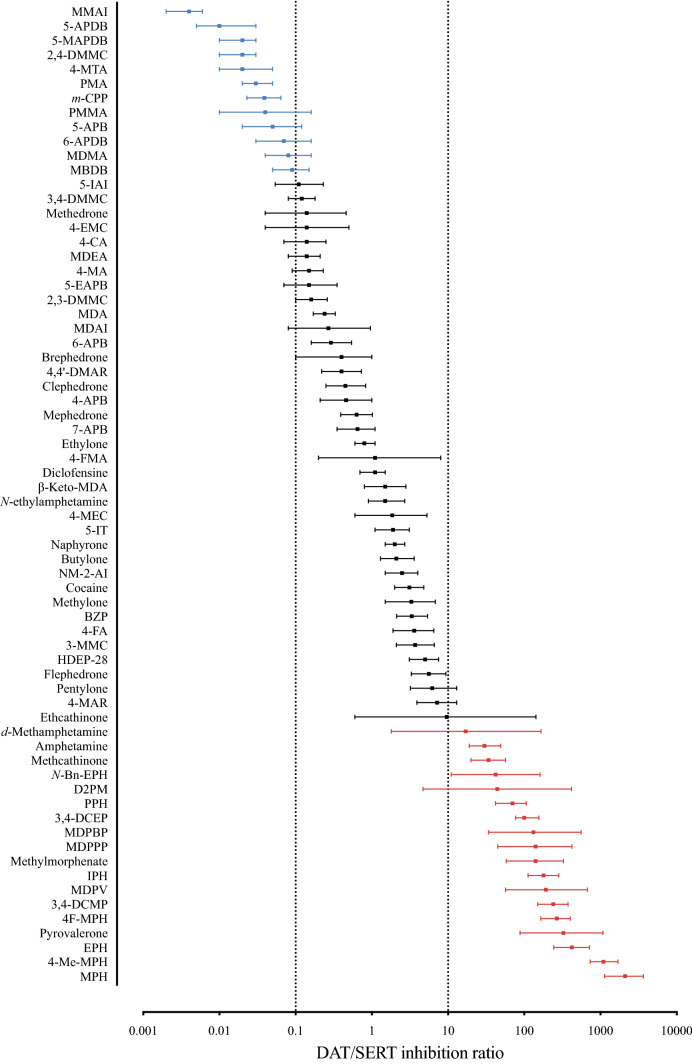
DAT vs*.* SERT selectivity of a variety of stimulants. Stimulants with low (< 0.1) DAT/SERT ratios are likely to induce entactogenic MDMA-like effects, while substances with a high (> 10) DAT/SERT ratio are associated with distinct psychostimulant effects and a high abuse potential. The DAT/SERT ratio is expressed as 1/DAT IC_50_: 1/SERT IC_50_. Full names of the substances and source of pharmacological data are provided in the supplementary information

**Fig. 2:**
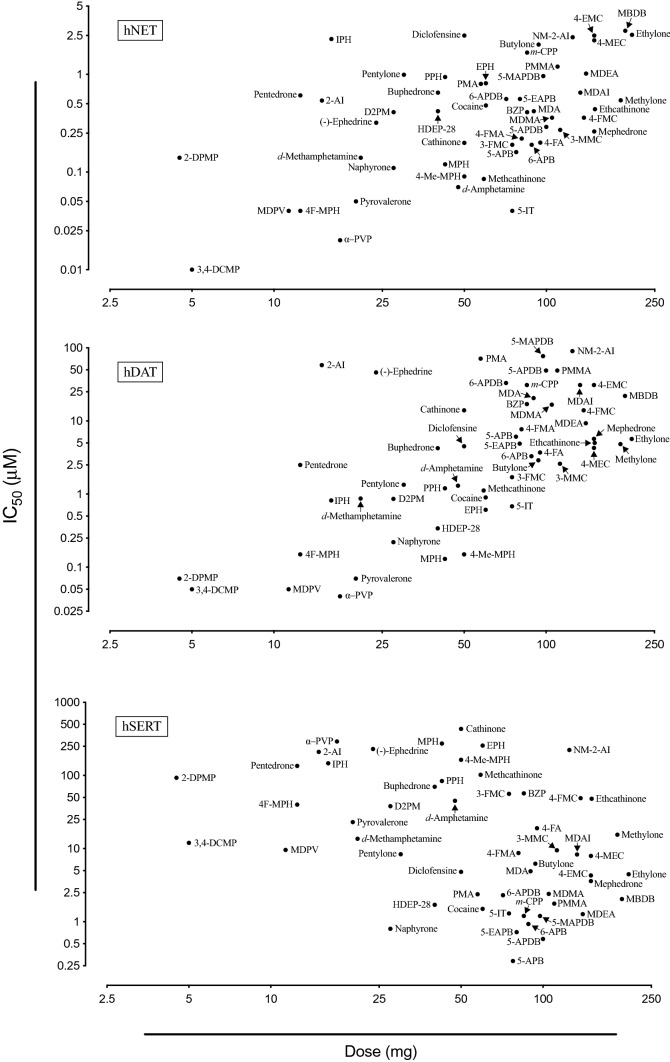
Correlation between reported clinical potencies and in vitro monoamine transporter inhibition of a variety of stimulants. Figure modified from (Luethi and Liechti 2018). Full names of the substances and source of pharmacological data are provided in the supplementary information

Monoaminergic stimulants, such as amphetamine, 3,4-methylenedioxymethamphetamine (MDMA), and cocaine, are among the most popular drugs of abuse. Other stimulants, such as methylphenidate and dextroamphetamine, are widely prescribed for the treatment of attention-deficit/hyperactivity disorder (ADHD). MDMA is currently being investigated as a prescription drug for the treatment of posttraumatic stress disorder (Amoroso and Workman 2016; Mithoefer et al. 2011, 2016; Sharma and Couture 2014). Stimulants modulate monoaminergic neurotransmission mainly by interacting with norepinephrine, dopamine, and serotonin (5-hydroxytryptamine [5-HT]) transporters (NET, DAT, and SERT, respectively), in addition to interacting with monoaminergic receptors and other targets. At monoamine transporters, monoaminergic stimulants act as either transporter inhibitors or substrates that mediate non-exocytotic monoamine efflux (Fleckenstein et al. 2000; Rothman and Baumann 2003; Sitte and Freissmuth 2015). Different selectivity (Fig. 1) and potency (Fig. 2) at the different transporters result in different pharmacological effects, different clinical potencies (i.e., the dose that is necessary to induce a psychoactive effect), and different abuse liabilities (Aarde and Taffe 2017; Gannon et al. 2018; Javadi-Paydar et al. 2018; Kuhar et al. 1991; Luethi and Liechti 2018; Ritz et al. 1987; Vandewater et al. 2015; Wee et al. 2005; Wee and Woolverton 2006). In rats, substances that are selective for DAT vs. SERT facilitate dose-dependent and abuse-related intracranial self-stimulation, indicating high abuse potential. In contrast, substances that are selective for the SERT vs. DAT depress dose-dependent intracranial self-stimulation (Suyama et al. 2019), indicating a lower risk of abuse. Repeated exposure to substances with similar activity at the DAT and SERT may sustain the expression of DAT-mediated abuse-related effects while developing tolerance to SERT-mediated abuse-limiting effects, thus resulting in a higher abuse potential (Suyama et al. 2019). The number of available designer stimulants is constantly increasing, and their use can cause various physiological complications and mood disturbances, which are discussed in the subsequent sections for the different classes of designer stimulants.

### Amphetamines

**Fig. 3:**
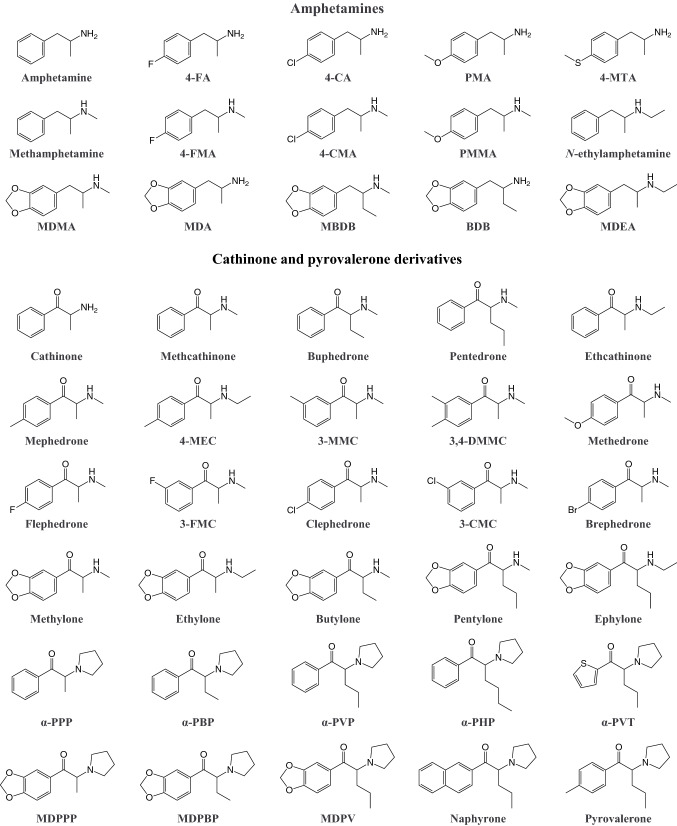
Examples of amphetamine, cathinone, and pyrovalerone derivatives. Full names of the substances are provided in the supplementary information

In addition to traditional amphetamines that are used both medically and recreationally, several amphetamine designer drugs without approved medical uses have become available. MDMA is by far the most popular amphetamine designer drug. It was first synthesized by Merck in 1912 as a precursor in a new chemical pathway, but it was not further investigated until many years later (Freudenmann et al. 2006). In the 1980s, MDMA started to be used in psychotherapy and became popular as a recreational drug under the street name “ecstasy,” which led to a ban of MDMA in most countries soon afterward (Freudenmann et al. 2006; Green et al. 2003). MDMA has slowly found its way back into psychotherapy as a promising agent for the treatment of posttraumatic stress disorder (Amoroso and Workman 2016; Mithoefer et al. 2016, 2011). Recently, various other, often ring-substituted amphetamine derivatives (Fig. 3) have gained increasing popularity as designer drugs, many of which were initially legally obtainable.

#### Mechanism of action of amphetamines

Most amphetamines are substrate-type monoamine releasers (Rothman and Baumann 2003; Simmler et al. 2013, 2014a; Sitte and Freissmuth 2015). In addition to potent effects at the NET, many amphetamines predominantly act at the DAT vs. SERT, resulting in greater reinforcing effects and higher abuse liability (Kuhar et al. 1991; Ritz et al. 1987; Wee et al. 2005; Wee and Woolverton 2006). In contrast, some amphetamines, including MDMA, have more pronounced effects at the SERT vs. DAT, resulting in an entactogenic effect profile and lower abuse liability (Baumann et al. 2000, 2012; Luethi et al. 2019a; Simmler et al. 2013). Para-substitution at the phenyl ring of amphetamines has been shown to shift their pharmacological profile toward more pronounced activity at the SERT vs. DAT (Luethi et al. 2018c, 2019b; Rickli et al. 2015a; Simmler et al. 2014a; Wee et al. 2005). In addition to their interactions with plasma membrane transporters, amphetamines are substrates at vesicular monoamine transporters (VMATs) and inhibit monoamine oxidases (Fleckenstein et al. 2007; Partilla et al. 2006; Sitte and Freissmuth 2015; Volz et al. 2007). Furthermore, amphetamine designer drugs have been reported to interact with various monoaminergic receptors, including serotonergic and adrenergic receptors, and trace amine-associated receptor 1 (TAAR1), which negatively modulates monoaminergic neurotransmission (Di Cara et al. 2011; Rickli et al. 2015a; Simmler et al. 2014a, 2016).

#### Adverse effects of amphetamines

Numerous studies have reported the adverse effects of amphetamine, lisdexamfetamine, and methamphetamine. Among amphetamine-derived designer drugs, MDMA is the best studied. For traditional amphetamines, mainly sympathomimetic adverse effects (e.g., anxiety, insomnia, headaches, mydriasis, bruxism, dry mouth, hyperthermia, hypertension, tachycardia, chest pain, palpitations, anorexia, nausea, vomiting, and abdominal pain) can be expected for newly emerged amphetamine-derived designer drugs (Carvalho et al. 2012; Derlet et al. 1989; Dolder et al. 2017; Heal et al. 2013; Vizeli and Liechti 2017; Wijers et al. 2017). Hyperthermia is a significant contributor to potentially severe adverse effects of amphetamines, including disseminated intravascular coagulation, renal failure, and rhabdomyolysis (Bingham et al. 1998; Carvalho et al. 2012; Cunningham 1997; Fahal et al. 1992; Ginsberg et al. 1970; Greene et al. 2003; Halachanova et al. 2001; Henry et al. 1992; Kendrick et al. 1977; Richards et al. 1999; Screaton et al. 1992; Vanden Eede et al. 2012). The uncoupling of oxidative phosphorylation in skeletal muscle through the activation of uncoupling protein 3 (UCP-3) and agonism at adrenergic receptors by norepinephrine release has previously been identified as an important contributor to MDMA-induced hyperthermia (Mills et al. 2003, 2004). Many adverse effects are similar for most amphetamines, but the prevalence of some events is higher for certain specific amphetamines. A comparison of the structures and pharmacological profiles of newly emerged amphetamine designer drugs with well-studied amphetamine derivatives helps to shed light on the likelihood of these specific adverse events. Hepatotoxicity is a potentially fatal adverse effect that has been associated with the use of amphetamines, and MDMA is the designer drug that has been most frequently linked to liver injury (Andreu et al. 1998; De Carlis et al. 2001; Ellis et al. 1996; Garbino et al. 2001; Jones et al. 1994; Kamijo et al. 2002). Different mechanisms may contribute to MDMA-induced hepatotoxicity, including monoamine release, hyperthermia, oxidative stress, impairments in the antioxidant response, mitochondrial dysfunction, and the formation of catechol metabolites by demethylenation (Carvalho et al. 2010, 2012). Cardiotoxicity is another potential complication of amphetamine use and largely attributable to sympathomimetic activation and additionally to secondary mechanisms, such as metabolic bioactivation and hyperthermia (Carvalho et al. 2012). The activation of 5-HT_2B_ receptors in cardiovascular tissues may potentially result in cardiac valvulopathy and is thus a concern for drugs that increase plasma 5-HT levels or directly activate 5-HT_2B_ receptors (Elangbam 2010; Elangbam et al. 2008; Huang et al. 2009; Roth 2007). Mild-to-moderate valvular heart disease has been observed in a population of heavy recreational MDMA users, and the 5-HT_2B_ receptor-mediated proliferation of cardiac valvular interstitial cells that was induced by MDMA was demonstrated in vitro (Droogmans et al. 2007; Setola et al. 2003). The MDMA metabolite 4-hydroxy-3-methoxymethamphetamine (HMMA) exhibits higher potency in stimulating vasopressin secretion; together with the excessive intake of hypotonic liquids and hyperthermia, it may cause potentially fatal hyponatremia, especially in female users likely because of effects of estrogen on vasopressin (Campbell and Rosner 2008; Fallon et al. 2002; Farah and Farah 2008; Forsling et al. 2001; Forsling et al. 2002; Ghatol and Kazory 2012; Hartung et al. 2002; Moritz et al. 2013; Rosenson et al. 2007; Simmler et al. 2011; Van Dijken et al. 2013). Monoamine depletion and reactive species contribute to the neurotoxicity of amphetamines (Carvalho et al. 2012). However, despite extensive research, the extent to which different amphetamines are neurotoxic remains largely unknown. Compared with amphetamine, an increase in serotonergic toxicity has been reported for the para-chlorinated derivative 4-chloroamphetamine, likely explained by highly potent serotonergic activity coupled with considerably potent dopaminergic activity (Colado et al. 1993; Fuller 1992; Johnson et al. 1990; Luethi et al. 2019b; Miller et al. 1986). However, unlike other halogenated stimulants, such as 4-fluoroamphetamine, 4-chloroamphetamine never achieved popularity as a designer drug, possibly because of its well-documented neurotoxicity. Nevertheless, the widely used 4-fluoroamphetamine has been associated with various mild-to-moderate adverse effects (e.g., agitation, severe headache, anxiety, confusion, tachypnea, hypertension, tachycardia, chest pain, electrocardiographic abnormalities, and nausea) and severe adverse effects (e.g., coma, convulsions, cerebral hemorrhage, inverted takotsubo cardiomyopathy, myocardial infarction, and fatalities following cardiac arrest) (Hondebrink et al. 2018). A detailed review of amphetamine toxicity, including toxicological pathways that involve the formation of reactive species, the depletion of antioxidants, and microglial activation, was previously published (Carvalho et al. 2012).

### Cathinone and pyrovalerone derivatives

Cathinone designer drugs are derivatives of the β-keto-amphetamine cathinone, an alkaloid that is found in the leaves of the *Catha edulis* plant. The large-scale recreational use of synthetic cathinones is a relatively new phenomenon, although several compounds have been known for a long time. For example, the first synthesis of 4-methylmethcathinone (mephedrone) was published in 1929 (Sanchez 1929). Several other synthetic cathinones have been investigated for their medical potential, mostly as antidepressant or anorectic agents, but only a few were ever marketed because of concerns about abuse (Canning et al. 1979; Cunningham 1963; Dal Cason et al. 1997; Seaton et al. 1961; Soroko et al. 1977; Valente et al. 2014). Pyrovalerone derivatives represent a subgroup of synthetic cathinones based on the structure of pyrovalerone, which was developed in the 1960s as a treatment option for lethargy, fatigue, and obesity (Gardos and Cole 1971). As a result of their initial misleading marketing as “bath salts”, synthetic cathinones are still often referred to by that term (Baumann et al. 2013). Currently, synthetic cathinones (Fig. 3) represent the largest group of designer stimulants that are monitored by the European Monitoring Center for Drugs and Drug Addiction (EMCDDA) (European Monitoring Centre for Drugs and Drug Addiction 2019).

#### Mechanism of action of cathinone and pyrovalerone derivatives

Similar to other monoaminergic stimulants, the psychoactive effects of synthetic cathinones are primarily mediated by interactions with monoamine transporters. Many cathinones are partially or fully effective substrate-type releasers at one or several monoamine transporters, but some compounds, such as pyrovalerone derivatives, are transporter inhibitors (Baumann et al. 2012; Eshleman et al. 2013, 2017; Luethi et al. 2018c; Mayer et al. 2016,2019a; Niello et al. 2019; Rickli et al. 2015a; Simmler et al. 2013). Mephedrone has additionally been shown to mediate monoamine release via organic cation transporter 3 (OCT3), indicating that cathinones target both high-affinity and low-affinity/high-capacity transporters (Mayer et al. 2019b). Similar to amphetamines, cathinone designer drugs also interact with several adrenergic and serotonergic receptors (Luethi et al. 2018c; Rickli et al. 2015a; Simmler et al. 2014a). Compared with amphetamines, however, cathinone designer drugs have been shown to interact less potently with TAAR1 and VMAT2 (Eshleman et al. 2013; Simmler et al. 2016). These less potent interactions at TAAR1 may result in a higher risk of cathinone dependence compared with amphetamines.

#### Adverse effects of cathinone and pyrovalerone derivatives

The use of synthetic cathinones has been associated with mainly sympathomimetic toxicity, which may manifest as agitation, tachycardia, hypertension and less frequently as lower levels of consciousness, hallucinations, hyponatremia, chest pain, palpitations, and nausea (Bäckberg et al. 2015c; Beck et al. 2015, 2016; Borek and Holstege 2012; Boulanger-Gobeil et al. 2012; Franzén et al. 2018; James et al. 2011; Ross et al. 2011, 2012; Umebachi et al. 2016; Wood et al. 2010). Rarely, severe adverse effects (e.g., seizures, significant peripheral organ damage, and rhabdomyolysis) have been reported (Bäckberg et al. 2015c; Beck et al. 2015, 2016; Borek and Holstege 2012; Boulanger-Gobeil et al. 2012; Franzén et al. 2018; Fröhlich et al. 2011; Penders et al. 2012; Ross et al. 2011, 2012). In vitro studies in neuronal, skeletal muscle, and hepatic cells indicated various cytotoxic mechanisms of synthetic cathinones, including mitochondrial dysfunction, glutathione depletion, oxidative stress, and apoptosis pathway activation, which are aggravated under hyperthermic conditions (Dias da Silva et al. 2019; Luethi et al. 2017, 2019b; Valente et al. 2016a, b, 2017a, b; Zhou et al. 2019). Unclear, however, is the extent to which these mechanisms contribute to clinical adverse effects of cathinones relative to sympathomimetic toxicity. Numerous cathinone-related fatalities have been reported (Adamowicz et al. 2014, 2016; Bäckberg et al. 2015c; Barrios et al. 2016; Beck et al. 2016; Busardò et al. 2015; Carbone et al. 2013; DeRoux and Dunn 2017; Eiden et al. 2013; Forrester 2012b; Kesha et al. 2013; Kudo et al. 2015; Lee et al. 2015; Liveri et al. 2016; Majchrzak et al. 2018; Marinetti and Antonides 2013; Maskell et al. 2011a; Nagai et al. 2014; Pearson et al. 2012; Pieprzyca et al. 2018; Potocka-Banas et al. 2017; Schifano et al. 2012; Sellors et al. 2014; Thirakul et al. 2017; Umebachi et al. 2016; Wood et al. 2010; Wright et al. 2013; Wyman et al. 2013; Zaami et al. 2018). Analytically confirmed cases of cathinone-related deaths were mainly attributed to hyperthermia, hypertension, cardiac arrest, and serotonin syndrome (Busardò et al. 2015; Zaami et al. 2018).

### Benzofuran and indole derivatives

**Fig. 4:**
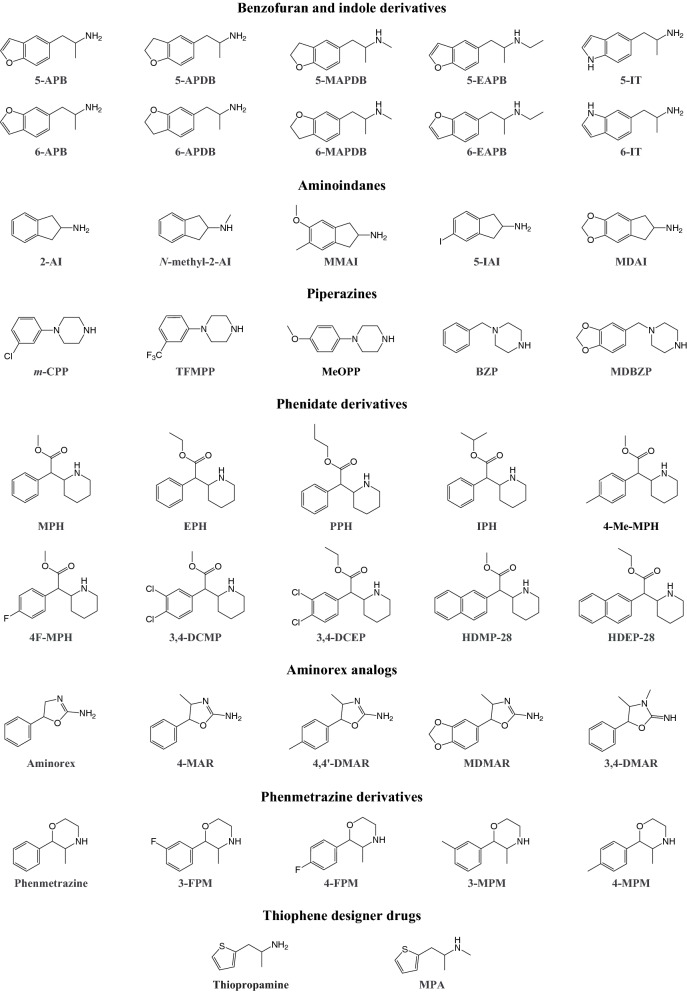
Examples of stimulant designer drugs and reference substances. Full names of the substances are provided in the supplementary information

Various analogs of MDMA and its metabolite 3,4-methylenedioxyamphetamine (MDA) have become available as designer drugs, in which a dihydrobenzofuran, benzofuran, or indole group replaces the benzodioxole group (Fig. 4). Some benzofuran designer drugs were originally investigated as part of a study that examined the role of ring oxygen atoms in interactions between MDA and monoamine transporters (Monte et al. 1993). The indole designer drugs 5-(2-aminopropyl)indole (5-IT, 5-API) and 6-(2-aminopropyl)indole (6-IT, 6-API) emerged from industrial research and are positional isomers of the psychedelic tryptamine α-methyltryptamine (αMT) (Hofmann and Troxler 1962).

#### Mechanism of action of benzofuran and indole derivatives

In addition to norepinephrine uptake inhibition, stimulant benzofuran and dihydrobenzofuran designer drugs have moderate-to-high selectivity in inhibiting 5-HT vs. dopamine uptake, often with substrate activity at the transporters (Monte et al. 1993; Rickli et al. 2015b). Furthermore, affinity at adrenergic, serotonergic, and histaminergic receptors, partial agonism at 5-HT_2A_ receptors, and partial to full agonism at 5-HT_2B_ receptors have been reported for these designer drugs (Dawson et al. 2014; Iversen et al. 2013; Rickli et al. 2015b). The indoles 5-IT and 6-IT are potent substrates at the NET, DAT, and SERT in rat synaptosomes (Marusich et al. 2016). The position of the alkylamine side chain is determining of DAT vs. SERT selectivity, with 5-IT having significantly (eightfold) more potent monoamine-releasing actions at the DAT vs. SERT in rat synaptosomes, whereas 6-IT is eightfold more selective for the SERT (Marusich et al. 2016). In human transporter-transfected cells, 5-IT has been shown to be a very potent inhibiter of norepinephrine uptake, but it did not significantly induce norepinephrine efflux at a single high concentration. However, it had substrate activity at the DAT and SERT (Luethi et al. 2018c). Additionally, 5-IT has affinity for adrenergic and serotonergic receptors and partially activates 5-HT_2A_ and 5-HT_2B_ receptors, which may result in additional perceptual psychedelic-like effects at high doses (Luethi et al. 2018c). Furthermore, 5-IT is an inhibitor of human monoamine oxidase (MAO)-A (Herraiz and Brandt 2014).

#### Adverse effects of benzofuran and indole derivatives

Benzofuran designer drugs may cause agitation, insomnia, headache, drowsiness, dry mouth, dry eyes, bruxism, hyperthermia, tachycardia, palpitations, nausea, diarrhea, hot flashes, clonus of the hands and feet, and psychological symptoms, including visual and auditory hallucinations, depression, anxiety, panic attacks, paranoia, and psychosis (Jebadurai et al. 2013; Nugteren-van Lonkhuyzen et al. 2015). A case of drug-induced psychosis with symptoms of self-harm, paranoia, and suicidal thoughts but unremarkable physical examination was reported with the analytically confirmed presence of 6-(2-aminopropyl)benzofuran (6-APB) in combination with metabolites of a synthetic cannabinoid and tetrahydrocannabinol (Chan et al. 2013). In addition to fatal intoxications that involve benzofurans combined with other designer drugs (Adamowicz et al. 2014; Elliott and Evans 2014), benzofuran toxicity was implicated as the cause of death in an accidental intoxication, in which 5-APB and presumptively a smaller amount of 5-(2-Aminopropyl)-2,3-dihydrobenzofuran (5-APDB) were detected as sole compounds in addition to alcohol (McIntyre et al. 2015a). Autopsy revealed white foam in the trachea, marked congestion and edema of the lungs, and congestive splenomegaly (McIntyre et al. 2015a). Benzofurans induce oxidative stress, disrupt mitochondrial function, and activate apoptosis cascades in vitro, but the in vivo relevance of these sequelae remain unclear (Roque Bravo et al. 2019). 5-IT has been linked to various sympathomimetic adverse effects, including extreme agitation, anxiety, confusion, insomnia, restlessness, hallucinations, seizures, tremors, dilated pupils without light reaction, hyperthermia, sweating, hypertension, tachycardia, arrhythmias, renal failure, myoclonus, muscle rigidity, rhabdomyolysis, and in some cases, serotonergic toxicity (Bäckberg et al. 2014; Coppola and Mondola 2013b; Katselou et al. 2015). Furthermore, 5-IT was involved in several intoxication cases with a fatal outcome within a time span of only a few months (Katselou et al. 2015; Kronstrand et al. 2013; Seetohul and Pounder 2013). Many of 5-IT-associated deaths have been attributed to cardiac arrest, to which 5-HT_2B_ receptor activation by 5-IT may have contributed (Katselou et al. 2015; Luethi et al. 2018c; Seetohul and Pounder 2013). In most of the fatal and non-fatal intoxication cases, additional substances have been detected. In some cases, the users reported to be unaware that they took 5-IT, because the products were mislabeled as 6-APB (Bäckberg et al. 2014; Kronstrand et al. 2013; Seetohul and Pounder 2013). Although the reported doses of 5-IT and 6-APB are similar, they differ in their selectivity for the dopaminergic vs. serotonergic system (Luethi et al. 2018c; Luethi and Liechti 2018; Rickli et al. 2015b). The extent to which mislabeling played a role in 5-IT intoxication remains unclear.

### Aminoindanes

Aminoindane designer drugs (Fig. 4) have become widely available when first-generation designer stimulants, including mephedrone, were finally placed under legal control (Pinterova et al. 2017; Sainsbury et al. 2011). Aminoindanes are conformationally restricted analogs of amphetamine that were originally investigated as bronchodilatory, analgesic, and anti-Parkinson agents, and subsequently as drugs with psychotherapeutic value (Pinterova et al. 2017; Solomons and Sam 1973). Some aminoindane designer drugs have been reported to be entactogens with lower serotonergic neurotoxicity relative to non-aminoindane entactogens (Johnson et al. 1990; Nichols et al. 1991). The desired psychoactive effects of aminoindane designer drugs include euphoria, the mild distortion of vision, time, and space, a greater intensity of perceptions and colors, empathy, and arousal (Coppola and Mondola 2013a; Corkery et al. 2013).

#### Mechanism of action of aminoindanes

Similar to amphetamines, aminoindane designer drugs are monoamine transporter substrates, with relevant affinity for adrenergic, dopaminergic, and serotonergic receptors (Iversen et al. 2013; Luethi et al. 2018c; Simmler et al. 2014b). Ring-substituted aminoindanes, such as 5,6-methylenedioxy-2-aminoindane (MDAI), 5-iodoaminoindane (5-IAI), and 5-methoxy-6-methyl-2-aminoindane (MMAI), are selective for the SERT vs*.* DAT (Luethi et al. 2018c; Simmler et al. 2014b). Potent actions of MDAI and 5-IAI on the NET result in an in vitro pharmacological profile that is similar to MDMA, suggesting similar entactogenic effects (Simmler et al. 2014b). MMAI acts as a selective 5-HT releaser with less pronounced effects on the NET, indicating that its effects are different from typical entactogens, such as MDMA (Luethi et al. 2018c). According to in vitro studies, the non-ring-substituted aminoindanes 2-aminoindane (2-AI) and *N*-methyl-2-AI are selective norepinephrine releasers and devoid of pharmacologically relevant DAT or SERT interactions (Luethi et al. 2018c; Simmler et al. 2014b).

#### Adverse effects of aminoindanes

Self-reported undesirable effects of aminoindane designer drugs include agitation, anxiety, panic attacks, headache, insomnia, hallucinations, and tachycardia (Coppola and Mondola 2013a). Three fatal cases were reported with confirmed MDAI intake, and serotonin syndrome could have been a factor that contributed to death (Corkery et al. 2013). The likelihood of the serotonergic toxicity of aminoindanes in humans has not been investigated, but signs of serotonin syndrome were reported for a high dose of MDAI in rats (Palenicek et al. 2016).

### Piperazines

Piperazine designer drugs (Fig. 4) have been widely sold as legal party pills or powders and appeared as pure substances or adulterants in pills that are sold as “ecstasy” because of their somewhat MDMA-like pharmacological profile, alone or combined (Baumann et al. 2005; Bossong et al. 2010; Lin et al. 2011; Sheridan et al. 2007; Wood et al. 2008). Various therapeutic drugs have a piperazine moiety, and some piperazine designer drugs have a history of medical use. For example, 1-benzylpiperazine (BZP) has been investigated as an antihelmintic agent and antidepressant, and meta-chlorophenylpiperazine (m-CPP) is an active metabolite of different antidepressants (Arbo et al. 2012; Schep et al. 2011). Other frequently used piperazine designer drugs include trifluoromethylphenylpiperazine (TFMPP), 1-(3,4-methylenedioxybenzyl)piperazine (MDBZP), and 4-methoxyphenylpiperazine (MeOPP).

#### Mechanism of action of piperazines

Piperazine designer drugs exert mixed effects at monoamine transporters. TFMPP and m-CPP are selective 5-HT vs. dopamine reuptake inhibitors (DAT/SERT ratio < 0.05), and m-CPP also inhibits norepinephrine uptake with potency that is similar to the inhibition of 5-HT uptake (Simmler et al. 2014b). Both substances bind to several serotonergic, adrenergic, dopaminergic, and histaminergic receptors with submicromolar or low micromolar affinity (Simmler et al. 2014b). In contrast, BZP is a selective NET inhibitor with relatively weak inhibition of dopamine and 5-HT uptake, without any potent affinity at monoamine receptors (Simmler et al. 2014b). BZP was also shown to be a DAT substrate in rat synaptosomes and human DAT-transfected cells, and m-CPP was reported to elicit 5-HT efflux in human SERT-transfected cells (Baumann et al. 2005; Simmler et al. 2014b). TFMPP mediated 5-HT efflux in rat synaptosomes but not in transfected cells at a single high concentration of 100 μM (Baumann et al. 2005; Simmler et al. 2014b). A combination of TFMPP and BZP was reported to closely mimic the effects of MDMA in rats (Baumann et al. 2005).

#### Adverse effects of piperazines

Adverse effects of piperazine designer drugs are mostly sympathomimetic, including agitation, insomnia, headaches, dizziness, dilated pupils, hyperthermia, tachycardia, nausea, urine retention, and inducible clonus (Arbo et al. 2012; Gee et al. 2005, 2008, 2010; Katz et al. 2016a; Kovaleva et al. 2008; Schep et al. 2011; Wilkins et al. 2008; Wood et al. 2008). In addition to sympathomimetic toxicity, dissociative symptoms, visual and auditory hallucinations, and psychological symptoms (e.g., short temper, confusion, anxiety, depression, and paranoia) have been associated with the use of piperazine designer drugs (Gee et al. 2008; Kovaleva et al. 2008; Schep et al. 2011; Wilkins et al. 2008; Wood et al. 2008). Furthermore, toxic seizures were frequently observed in patients who were admitted to the emergency department after the use of BZP-containing party pills. Although there seems to be a trend toward higher concentrations being more frequently associated with seizures, they may also occur at low doses (Gee et al. 2005, 2008). Other severe adverse effects of BZP include hyponatremia, severe combined metabolic and respiratory acidosis, hepatic injury, renal failure, disseminated intravascular coagulation, and rhabdomyolysis (Gee et al. 2010; Katz et al. 2016a). A case of severe hyperthermia with resultant multi-organ failure and a case of hyponatremia that led to fatal brain edema were reported for the concomitant use of piperazine designer drugs and MDMA (Balmelli et al. 2001; Gee et al. 2010). The contribution of these individual compounds to the observed clinical manifestations remains unclear, but piperazines and MDMA may elicit additive or synergistic toxicity. In vitro, piperazine designer drugs have been reported to upregulate key enzymes of cholesterol biosynthesis, induce oxidative stress, disrupt mitochondrial function, and activate apoptosis pathways, all of which may potentially contribute to clinical toxicity (Arbo et al. 2016a, b; Dias da Silva et al. 2017; Dias-da-Silva et al. 2015; Majrashi et al. 2018).

### Phenidate derivatives

Derivatives of the piperidine prescription drug methylphenidate have appeared as designer drugs (Fig. 4), with substitutions at the phenyl ring and different lengths of the carbon side chain (Luethi et al. 2018b). Similar to methylphenidate, phenidate derivatives may be used to induce euphoria or as cognitive enhancers (Ho et al. 2015; Lüthi and Liechti 2019). Various methylphenidate-based designer drugs originated from drug development efforts and later appeared on the recreational drug market as pure compounds or in the form of branded products (Bailey et al. 2015; Deutsch et al. 1996; Ho et al. 2015; Markowitz et al. 2013; Misra et al. 2010). When insufflated, the pharmacological and subjective-effect profile of methylphenidate is similar to cocaine, and phenidate derivatives may, therefore, be used as substitutes for cocaine (Vogel et al. 2016).

#### Mechanism of action of phenidate derivatives

Similar to methylphenidate, methylphenidate-based designer drugs act as potent NET and DAT inhibitors that are devoid of substrate activity (Luethi et al. 2018b; Simmler et al. 2014b). Some less potent interactions with the SERT and adrenergic and serotonergic receptors have been reported but are not likely to play a relevant role in the psychoactive actions of most phenidate derivatives (Luethi et al. 2018b).

#### Adverse effects of phenidate derivatives

Adverse effects of phenidate derivatives are similar to amphetamines and include agitation, anxiety, hypertension, tachycardia, and palpitations (Bailey et al. 2015). Because of their relatively slow onset of action when taken orally, the nasal insufflation or injection of phenidate derivatives is common, especially in heavy users. Nasal pain and septum perforations after insufflation and infections after intravenous injection may occur (Ho et al. 2015; Lafferty et al. 2016; Parks et al. 2015). The rapid onset of action after nasal or intravenous use, combined with the marked DAT vs. SERT selectivity of phenidate derivatives, has been linked to a higher risk of addiction (Luethi et al. 2018b). In several cases, phenidate derivatives have been analytically confirmed post-mortem, in which ethylphenidate is the most frequently detected compound (Krueger et al. 2014; Maskell et al. 2016; Parks et al. 2015; Shoff et al. 2019). Many decedents had a history of heroin use, and intravenous injection was a common route of administration. In addition to phenidate derivatives, other drugs, including benzodiazepines and opioids, have been detected in most fatal cases (Krueger et al. 2014; Maskell et al. 2016; Parks et al. 2015).

### Aminorex analogs

Various analogs of the anorectic agent aminorex have become available as designer drugs (Fig. 4). Aminorex was first marketed as an over-the-counter appetite suppressant in parts of Europe in the 1960s, but it was withdrawn a few years later because of an epidemic of chronic pulmonary hypertension that was associated with many fatalities (Maier et al. 2018a). Aminorex analogs that have found their way onto the designer drug market include 4-methylaminorex (4-MAR) and 4,4′-dimethylaminorex (4,4′-DMAR), the reported effects of which include euphoria, mental and physical stimulation, sociability, empathy, arousal, and changes in visual perception (European Monitoring Centre for Drugs and Drug Addiction 2015; Glanville et al. 2015; Loi et al. 2017). A comprehensive review of the history of aminorex use and the emergence of its designer drug analogs was recently published (Maier et al. 2018a).

#### Mechanism of action of aminorex analogs

In human transporter-transfected cells, 4,4′-DMAR is a potent inhibitor of norepinephrine, dopamine, and 5-HT reuptake. 4-MAR has similarly potent dopamine and norepinephrine reuptake properties as 4,4′-DMAR, but 5-HT uptake inhibition is less pronounced compared with its para-methylated counterpart (Maier et al. 2018b; Rickli et al. 2019). Aminorex and its derivative 4-MAR mediate norepinephrine and dopamine efflux in rat synaptosomes, with weak substrate activity at the SERT (Brandt et al. 2014; Rothman et al. 2001). 4,4′-DMAR and 3′,4′-methylenedioxy-4-methylaminorex (MDMAR) induce norepinephrine, dopamine, and 5-HT efflux in rat synaptosomes (Brandt et al. 2014; McLaughlin et al. 2015). Dynamic superfusion experiments revealed the substrate activity of 4,4′-DMAR at human monoamine transporters (Maier et al. 2018b). In human transporter-transfected cells that were preloaded with monoamines and exposed to drugs at a single high concentration (100 μM), only dopamine efflux was observed for 4,4′-DMAR, and dopamine and 5-HT efflux was observed for 4-MAR (Rickli et al. 2019). In addition to interactions with plasmalemmal transporters, 4,4′-DMAR has been shown to inhibit human VMAT2-mediated dopamine uptake (Maier et al. 2018b). In addition to their primary effects on transporters, minor interactions with serotonergic 5-HT_2C_ and adrenergic α_2A_ receptors have been described for 4-MAR, and low affinity at 5-HT_2A_ and 5-HT_2C_ receptors has been described for 4,4′-DMAR (Maier et al. 2018b; Rickli et al. 2019).

#### Adverse effects of aminorex analogs

Adverse effects of aminorex designer drugs that have been reported by users on various Internet discussion platforms include agitation, dysphoria, insomnia, amnesia, panic attacks, psychosis, hallucinations, facial spasms, dilated pupils, foaming at the mouth, dry mouth, jaw clenching, elevations of body temperature, sweating, elevations of heart rate, nausea, and restless legs (Glanville et al. 2015; Loi et al. 2017; Maier et al. 2018a). Pulmonary hypertension (i.e., the adverse effect that led to the removal of aminorex from the market) has been associated with the recreational use of 4-MAR (Gaine et al. 2000). Designer drug analogs of aminorex have been analytically confirmed in several drug-related deaths (Cosbey et al. 2014; Davis and Brewster 1988; European Monitoring Centre for Drugs and Drug Addiction 2015). Although other substances were present in most fatal cases, 4,4′-DMAR was mentioned to be the cause of death or to have played a contributory role in several of these fatalities (European Monitoring Centre for Drugs and Drug Addiction 2015). Brain edema, seizures, hyperthermia, respiratory and cardiac arrest, and internal bleeding were all listed as adverse events or autopsy findings that were associated with the use of 4,4′-DMAR (European Monitoring Centre for Drugs and Drug Addiction 2015).

### Phenmetrazine derivatives

Phenmetrazine is a reinforcing stimulant, which was previously used as an appetite suppressant before it was eventually withdrawn from the market (Chait et al. 1987). Phenmetrazine-derived designer drugs (Fig. 4) represent a relatively understudied class of drugs, among which 3-fluorophenmetrazine (3-FPM) use appears to be the most widespread.

#### Mechanism of action of phenmetrazine derivatives

Like the parent compound, ring-fluorinated derivatives of phenmetrazine are substrates at the NET and DAT, with minor substrate activity at the SERT (Mayer et al. 2018; Rothman et al. 2002). Ring-methylated phenmetrazine derivatives were reported to have greater potency at the SERT, in addition to activity at the NET and DAT (McLaughlin et al. 2018). Para-substituted compounds were shown to have the greatest serotonergic effects among the phenmetrazine derivatives, similar to ring-substituted amphetamine and cathinone designer drugs (Luethi et al. 2019b; Rickli et al. 2015a).

#### Adverse effects of phenmetrazine derivatives

Based on their mechanism of action, phenmetrazine designer drugs are expected to elicit stimulatory toxicity that is similar to amphetamines. A series of non-fatal intoxications that involved 3-FPM were reported within the Swedish STRIDA project, mostly with sympathomimetic adverse effects (Bäckberg et al. 2016). However, polydrug intoxication prevented attribution of the observed effects to 3-FPM, underscored by the fact that the clinical features included some oppositional effects (e.g., both miotic and dilated pupils and both hypertension and hypotension) (Bäckberg et al. 2016). Nevertheless, the authors of this case series suggested that 3-FPM is a harmful compound, as one-third of patients presented severe adverse events (Bäckberg et al. 2016). In addition to other polydrug intoxications that involve 3-FPM (Benesch and Iqbal 2018; Ellefsen et al. 2017), a case of severe kidney injury and limb ischemia that were associated with intravenous 3-FPM use was reported (Fawzy et al. 2017). The authors of the latter case report hypothesized that the intravenous use of 3-FPM resulted in severe vasoconstriction, possibly with concomitant infection, and caused widespread ischemia (Fawzy et al. 2017).

### Thiophene designer drugs

Various analogs of amphetamines and cathinones with a thiophene group that replaces the phenyl ring have appeared as designer drugs (Fig. 4). Some of the thiophene designer drugs were first described in the 1940s and elicited effects that were reported to be comparable to their phenyl ring analogs (Alles and Feigen 1941; Blicke and Burckhalter 1942). To date, most pharmacological studies and toxicological reports involve methiopropamine (MPA), the thiophene analog of methamphetamine.

#### Mechanism of action of thiophene designer drugs

MPA is a quasi-equipotent inhibitor of norepinephrine and dopamine uptake and was reported to interact with various serotonergic, adrenergic, dopaminergic, *N*-methyl-d-aspartate (NMDA), and sigma-1 receptors (Iversen et al. 2013).

#### Adverse effects of thiophene designer drugs

MPA use has been associated with significant acute toxicity and psychotic, cardiovascular, and gastrointestinal symptoms, including agitation, anxiety, confusion, a lower level of consciousness, insomnia, visual hallucinations, elevations of creatine kinase, tachycardia, palpitations, chest tightness, nausea, and vomiting (Daveluy et al. 2016; Lee et al. 2014; White et al. 2019). However, for most intoxication cases, the use of multiple substances was reported, and the extent to which MPA contributed to the reported adverse effects remains unclear. A death from isolated MPA use was described, in which cardiac arrhythmia that induced cardiovascular collapse was named as the probable cause of death (Anne et al. 2015). Dopaminergic neurodegeneration and myocardial, renal, and gastrointestinal damage were observed in mice that were exposed to MPA (Foti et al. 2019; Nguyen et al. 2019).

### Miscellaneous stimulants

Several designer drugs have appeared that do not belong to any classes that are discussed in the previous sections. Any substance that interacts with monoamine transporters may potentially be sold as a stimulant designer drug, even if it is not or only remotely chemically related to the widely used stimulant classes. One example of such a substance is the potent NET, DAT, and SERT blocker diclofensine, a tetrahydroisoquinoline derivative that was originally developed as an antidepressant (Luethi et al. 2018a).

## Sedatives

### Synthetic opioids

**Fig. 5:**
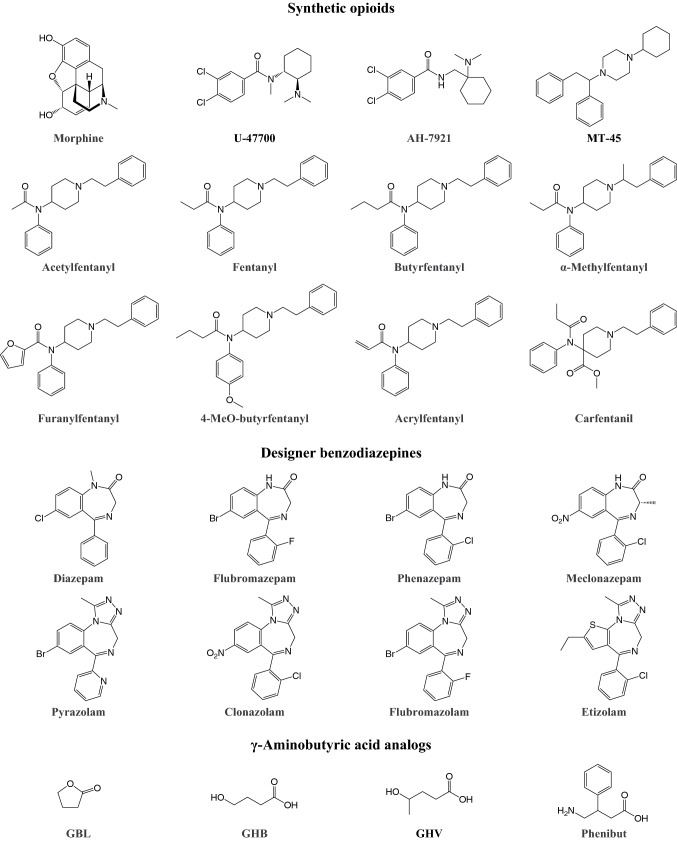
Examples of sedative designer drugs and reference substances for comparison. Full names of the substances are provided in the supplementary information

While being essential for pain treatment, the non-medical use of opioids has been a public health threat for centuries and includes the recreational use of illegal substances, the abuse of prescription medications, and drug adulteration with non-pharmaceutical opioids (Armenian et al. 2018b). Opioids induce euphoria, anxiolysis, feelings of relaxation, and drowsiness (Suzuki and El-Haddad 2017). Repeated use leads to the development of dependence. In recent years, the growing prevalence of non-pharmaceutical fentanyl, highly potent designer fentanyls, and other novel synthetic opioids (Fig. 5) has critically contributed to the opioid crisis, particularly in the United States (Daniulaityte et al. 2017; Denton et al. 2008; Lucyk and Nelson 2017; Peterson et al. 2016; Rudd et al. 2016; Scholl et al. 2018; Seth et al. 2018). Notably, fentanyl-type substances are often detected in fatalities that are presumably associated with intravenous heroin use (Gladden et al. 2019). The higher potency of fentanyl and its analogs compared with classic heroin results in a higher risk of overdose, particularly when they are mistaken for heroin. Fentanyl itself was first synthesized in 1960 and has become essential and widely used for intraoperative analgesia and in the form of transdermal patches for the management of chronic pain (Stanley 2014). Following its medical approval, reports of fentanyl misuse among clinicians and subsequently patients began to emerge, and several fentanyl analogs appeared on the illicit market (Armenian et al. 2018b; Suzuki and El-Haddad 2017). In Europe’s drug market, 49 new synthetic opioids were detected between 2009 and 2018, 34 of which are fentanyl derivatives (European Monitoring Centre for Drugs and Drug Addiction 2019).

#### Mechanism of action of synthetic opioids

Novel fentanyl analogs and other synthetic opioids interact with G protein-coupled opioid receptors in the brain and spinal cord as partial to full agonists at μ-, δ-, and κ-opioid receptor subtypes, with selectivity for the μ-opioid receptor (Armenian et al. 2018b; Baumann et al. 2018; Codd et al. 1995; Maguire et al. 1992; Prekupec et al. 2017). Multiple lines of evidence indicate that agonism at μ-opioid receptors drives the main pharmacological effects of opioids, including euphoria, analgesia, respiratory depression, and the development of dependence (Charbogne et al. 2014; Kieffer 1999). A detailed overview of signaling mechanisms and behavioral effects of opioid receptor activation is provided elsewhere (Al-Hasani and Bruchas 2011). In vitro pharmacological profiling appears to be only a limited predictor of the clinical potency of opioids (Baumann et al. 2018). However, rodent tail flick tests suggest mostly distinctively greater potencies of novel synthetic opioids compared with morphine (Armenian et al. 2018b). For example, the potency of fentanyl is reported to be 50- to 200-fold higher than morphine, and the potency of carfentanil is reported to be approximately 10,000 times higher than morphine (Armenian et al. 2018b; Concheiro et al. 2018; Suzuki and El-Haddad 2017). Several prescription opioids inhibit the NET and SERT and interact with 5-HT_2_ receptors (Codd et al. 1995; Rickli et al. 2018). Fentanyl has affinity for 5-HT_1A_ and 5-HT_2A_ receptors but is devoid of relevant monoamine transporter interactions (Barann et al. 2015; Rickli et al. 2018). No data are currently available on monoamine transporter interactions of designer opioids.

#### Adverse effects of synthetic opioids

Adverse effects of novel synthetic opioids include typical symptoms of opioid overdose, such as dizziness, a lower level of consciousness, miosis, central nervous system depression, respiratory depression, pulmonary edema, hypoxia, bradycardia, pruritus, nausea, vomiting, constipation, and also such symptoms as agitation, hypertension, and tachycardia (Armenian et al. 2017, 2018b; Bäckberg et al. 2015b; Domanski et al. 2017; Helander et al. 2014, 2016, 2017a; Jones et al. 2017; Müller et al. 2019; Schneir et al. 2017; Siddiqi et al. 2015; Wilde et al. 2020). Pulmonary edema, acute lung injury, diffuse alveolar hemorrhage, renal insufficiency, and rhabdomyolysis were also reported in patients who presented with designer opioid intoxication (Cole et al. 2015; Helander et al. 2016, 2017a). The synthetic opioid 1-cyclohexyl-4-(1,2-diphenylethyl)piperazine (MT-45) has been associated with bilateral hearing loss and hearing disturbances, with likely irreversible and pronounced sensorineural hearing impairment in one case (Helander et al. 2014). Furthermore, acute skin and hair symptoms followed by severe delayed eye complications were reported in patients with confirmed MT-45 use; cataract surgery was required in two of these patients (Helander et al. 2017b). Remaining unclear, however, is whether the aforementioned complications are solely attributable to MT-45 toxicity. Serotonergic toxicity is one adverse effect that needs to be considered for opioid designer drugs when combined with other serotonergic agents (Baldo 2018; Rickli et al. 2018). Similar to traditional opioids, withdrawal from designer opioids may result in physiological and psychological distress (Siddiqi et al. 2015). Numerous fatalities have been attributed to either designer opioids alone or designer opioids combined with other psychoactive substances (Coopman et al. 2016a, b; Cunningham et al. 2016; Dussy et al. 2016; Dwyer et al. 2018; Elliott et al. 2016; Fels et al. 2017, 2019; Fort et al. 2016; Garneau et al. 2019; Gillespie et al. 1982; Guerrieri et al. 2017; Helander et al. 2017a; Karinen et al. 2014; Koch et al. 2018; Kriikku et al. 2019; Kronstrand et al. 2014; Krotulski et al. 2018; Martucci et al. 2018; McIntyre et al. 2015b, 2016, 2017; Mohr et al. 2016; Nash et al. 2019; Ojanperä et al. 2006; Papsun et al. 2016; Partridge et al. 2018; Poklis et al. 2016; Richeval et al. 2019; Ruan et al. 2016; Sofalvi et al. 2017; Staeheli et al. 2016; Swanson et al. 2017; Takase et al. 2016; Vorce et al. 2014; Yonemitsu et al. 2016). Frisoni and colleagues recently published an overview of opioid-related fatalities that were attributed to synthetic opioids (Frisoni et al. 2018). In addition to central nervous system and respiratory depression, chest wall rigidity after intravenous use could be a cause of death in synthetic opioid overdose cases (Burns et al. 2016). The competitive μ-opioid receptor antagonist naloxone rapidly reverses central and peripheral effects of opioids and is thus an effective antidote for opioid toxicity (Armenian et al. 2018b). The initial care of patients who are intoxicated with designer opioids should focus on airway protection and maintaining breathing and circulation (Armenian et al. 2018b). Naloxone should be administered as soon as possible (Armenian et al. 2018b; Kim and Nelson 2015).

### Designer benzodiazepines

In 1960, chlordiazepoxide became the first of several medically approved benzodiazepines that today represent a widely prescribed class of drugs for the treatment of psychiatric and neurological conditions, particularly insomnia and anxiety disorders (Longo and Johnson 2000; Sternbach 1979). Benzodiazepine abuse is frequent. The main reasons for such abuse are to facilitate sleep, cope with stress, ease effects of stimulants, self-treat withdrawal symptoms, and get high (Kapil et al. 2014; Vogel et al. 2013; Zawilska and Wojcieszak 2019). Benzodiazepines have limited potential as euphoriants when administered alone. When taken in combination with opioids, however, benzodiazepines appear to enhance the euphoric effects of opioid use (Jones et al. 2012). Since 2007, several benzodiazepine designer drugs (Fig. 5) have become available, some of which are precursors or metabolites of prescription benzodiazepines and are approved for medical use in other countries (Bäckberg et al. 2019; Manchester et al. 2018). Effects of designer benzodiazepines reported on internet forums resemble those of prescription benzodiazepines (El Balkhi et al. 2020). Chronological overviews of the appearance of benzodiazepine designer drugs on the recreational drug market were recently published (Manchester et al. 2018; Moosmann and Auwärter 2018).

#### Mechanism of action of designer benzodiazepines

The mechanism of action of most benzodiazepine designer drugs currently remains understudied. In silico experiments suggest that they mediate their effects through interactions at γ-aminobutyric acid-A (GABA_A_) receptors such as prescription benzodiazepines (Waters et al. 2018). GABA_A_ receptors are ion channels that consist of pentamers of different subunit compositions, responding to the inhibitory neurotransmitter GABA. Benzodiazepines enhance the effects of GABA as positive allosteric modulators by binding to a receptor site that is different from the binding site of GABA (Manchester et al. 2018; Moosmann and Auwärter 2018).

#### Adverse effects of designer benzodiazepines

Despite their depressive actions on central nervous system function and respiration, the isolated use of benzodiazepines is rarely fatal. However, in reported intoxication cases, designer benzodiazepines have mostly been detected in combination with other psychoactive substances, such as stimulants or depressants (Bäckberg et al. 2019). The concurrent use of benzodiazepines and other depressants, such as opioids and alcohol, may produce prolonged and potentially fatal respiratory depression (Jones et al. 2012; Zawilska and Wojcieszak 2019). Reported adverse effects of isolated benzodiazepine designer drugs are typical for a sedative-hypnotic toxidrome but may include atypical symptoms in some cases, such as agitation, hyperthermia, and tachycardia (Bäckberg et al. 2019; Carpenter et al. 2019; Zawilska and Wojcieszak 2019). The recent review by Zawilska and Wojcieszak mentioned the following adverse effects of designer benzodiazepines: fatigue, impairment of thinking, confusion, dizziness, drowsiness, lethargy, amnesia, blurred vision, slurred speech, palpitations, and muscle weakness, as well as auditory and visual hallucinations, delirium, seizures, deep sleep, and coma at high doses (Zawilska and Wojcieszak 2019). The chronic use of designer benzodiazepines may also lead to the development of tolerance and dependence (Zawilska and Wojcieszak 2019). Withdrawal symptoms, such as anxiety, panic attacks, restlessness, insomnia, seizures, and life-threatening convulsions, may follow the abrupt cessation of chronic designer benzodiazepines use (Andersson and Kjellgren 2017; Zawilska and Wojcieszak 2019). Designer benzodiazepines have been reported to contribute to numerous deaths (Bailey et al. 2010; Crichton et al. 2015; Domingo et al. 2017; Karinen et al. 2014; Koch et al. 2018; Liveri et al. 2016; Maskell et al. 2011b; Papsun et al. 2016; Partridge et al. 2018; Shearer et al. 2015; Tanaka et al. 2011a, b). In a few cases, the cause of death was solely attributed to the designer benzodiazepine phenazepam (Crichton et al. 2015; Shearer et al. 2015) or etizolam (Carpenter et al. 2019). A more detailed summary of benzodiazepine-related fatalities was recently published (Zawilska and Wojcieszak 2019).

### γ-Aminobutyric acid analogs

γ*-*Hydroxybutyrate (GHB) is a short-chain fatty acid analog of the inhibitory neurotransmitter GABA. It has become popular among drug users because of its ability to induce feelings of euphoria and relaxation, reduce social anxiety, and increase sexual drive (Brennan and Van Hout 2014; Brown et al. 2011). Although it is an endogenous compound and its sodium salt is approved as a prescription drug against narcolepsy, GHB and its metabolic precursors (e.g., γ-butyrolactone [GBL] and 1,4-butanediol [1,4-BD]) are often referred to as designer drugs because of their widespread illicit production in clandestine laboratories (Brennan and Van Hout 2014; Fuller et al. 2004). Other structural analogs of GABA that have become available as designer drugs (Fig. 5) include the 4-methyl-substituted GHB derivative γ*-*hydroxyvaleric acid (GHV) and 4-amino-3-phenyl-butyric acid (phenibut) (Carter et al. 2005; Owen et al. 2016).

#### Mechanism of action of GABA analogs

Metabotropic G protein-coupled GABA_B_ receptors are the primary targets of designer drug analogs of GABA (Brennan and Van Hout 2014; Carai et al. 2001; Lapin 2001). Other postulated mechanisms of action include high-affinity binding to receptor sites that are distinct from the GABA_B_ receptor (i.e., GHB receptors), binding to specific GABA_A_ receptor subtypes, and monoaminergic modulation (Bay et al. 2014; Carter et al. 2009; Crunelli et al. 2006; Lapin 2001; Wood et al. 2011).

#### Adverse effects of GABA analogs

Comprehensive reviews of the potential clinical complications of the use of GHB and its metabolic precursors are provided elsewhere (Busardò and Jones 2015; Schep et al. 2012). The subjective benefits of GHB and its analogs outweigh adverse events only over a narrow range of doses. Adverse events include a lower level of consciousness, hypothermia, respiratory depression, aspiration, bradycardia, gastrointestinal upset, and nonsedative adverse effects, such as agitation, seizures, and myoclonus (Busardò and Jones 2015; Isoardi et al. 2020; Liakoni et al. 2016; McCabe et al. 2019; Schep et al. 2012; Zvosec and Smith 2005). These adverse effects typically have a relatively short duration and are usually managed with supportive care (Busardò and Jones 2015; Schep et al. 2012; Wood et al. 2011). GABA_B_ and monocarboxylate transporter inhibitors have been proposed as potential treatment options for GHB-induced respiratory depression (Morse et al. 2012). GHB and its analogs are associated with the rapid development of tolerance. Abrupt cessation after regular use may trigger a potentially life-threatening withdrawal syndrome that can manifest as agitation, anxiety, confusion, disorientation, paranoia, aggression, insomnia, auditory and visual hallucinations, tremors, sweating, hypertension, and tachycardia (Busardò and Jones 2015; Owen et al. 2016; Schep et al. 2012; Wood et al. 2011). Benzodiazepines appear to be the treatment of choice for withdrawal from GHB and its analogs (Busardò and Jones 2015; Schep et al. 2012; Wood et al. 2011). Zvosec and colleagues reported a series of 226 GHB-associated deaths, 213 of which were attributed to cardiorespiratory arrest and 13 of which were attributed to fatal accidents (Zvosec et al. 2011). In approximately one-third of these fatal cases, GHB was the sole toxicant detected (Zvosec et al. 2011). Similar findings were reported by Corkery and colleagues, who reported a series of 159 GHB and GBL-associated fatalities (Corkery et al. 2015). The co-ingestion of opioids increases the depressant toxicity of GHB, and stimulant intake does not appear to prevent GHB toxicity (Knudsen et al. 2010).

## Dissociatives

### Arylcyclohexylamine and diarylethylamine designer drugs

**Fig. 6:**
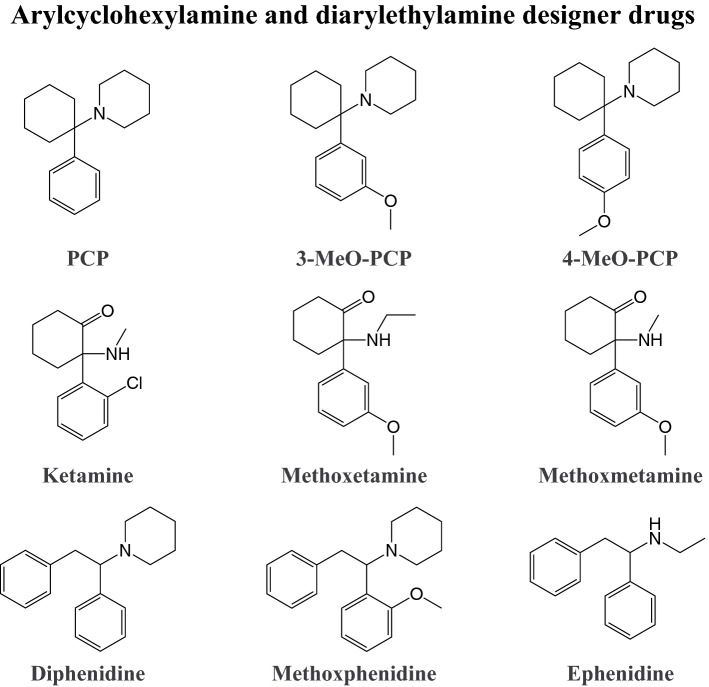
Examples of dissociative designer drugs and reference substances for comparison. Full names of the substances are provided in the supplementary information

Dissociative agents are appreciated in medicine because of their unique pharmacological effects. These pharmacological effects, however, are also popular among recreational drug users. The dissociative anesthetic ketamine produces analgesia without cardiovascular or respiratory depression at doses that produce anesthesia, a feature that is not shared by other common anesthetics (Li and Vlisides 2016). Furthermore, ketamine induces rapid and sustained antidepressant actions at a single sub-anesthetic dose and has become a widely abused recreational drug because of its dissociative effects, including sensory and tactile distortions, euphoria, and depersonalization (Li and Vlisides 2016; Zanos and Gould 2018). Ketamine was first synthesized in 1962 as a short-acting anesthetic with lower potency in producing emergence delirium compared with the structurally similar phencyclidine (PCP). PCP was developed before ketamine as a promising dissociative anesthetic, but its use in humans and animals was discontinued because of its unfavorable side effects (Domino 1980). Various similar substances have been clinically investigated. Dissociatives began to appear on the illicit drug market in the late 1960s (Morris and Wallach 2014). Today, several dissociative designer drugs (Fig. 6) are available, mostly arylcyclohexylamines (e.g., ketamine and PCP) and diarylethylamines. Morris, Wallach, and Brandt previously published comprehensive overviews of the history, availability, and use of several arylcyclohexylamine and diarylethylamine designer drugs and other dissociative agents (Morris and Wallach 2014; Wallach and Brandt 2018a, b).

#### Mechanism of action of arylcyclohexylamine and diarylethylamine designer drugs

Similar to ketamine and PCP, dissociative arylcyclohexylamine and diarylethylamine designer drugs act as relatively selective noncompetitive antagonists at ionotropic glutamatergic NMDA receptors. Their NMDA receptor affinity is strongly correlated with their clinical potency in inducing dissociative effects in vivo (Anis et al. 1983; Morris and Wallach 2014; Wallach et al. 2016). Some dissociative designer drugs also moderately inhibit the reuptake of norepinephrine and dopamine, whereas others have appreciable affinity for the SERT (Luethi et al. 2018a; Roth et al. 2013; Wallach et al. 2016). Binding affinity at various receptors, including α adrenergic, serotonergic, histaminergic, cholinergic, opioidergic, and sigma receptors, has been reported for arylcyclohexylamines and diarylethylamines (Luethi et al. 2018a; Roth et al. 2013; Wallach et al. 2016). Summarized, NMDA receptor antagonism mainly mediates the dissociative effects of arylcyclohexylamines and diarylethylamines, and interactions with other pharmacological targets may modify the activity of different compounds.

#### Adverse effects of arylcyclohexylamine and diarylethylamine designer drugs

Adverse effects of dissociative arylcyclohexylamine and diarylethylamine designer drugs resemble adverse effects of traditional dissociatives, including agitation, confusion, disorientation, dissociation, hallucinations, amnesia, nystagmus, slurred speech, diaphoresis, hypertension, tachycardia, renal deficiency, nausea, ataxia, and muscle rigidity (Bäckberg et al. 2015a; Dunlop et al. 2019; Gerace et al. 2017; Helander et al. 2015; Hofer et al. 2012, 2014; Johansson et al. 2017; Shields et al. 2012; Thornton et al. 2017; Ward et al. 2011; Wood et al. 2012; Zawilska 2014; Zidkova et al. 2017). In severe cases, dissociative designer drugs may potentially cause neurological impairment, manifested as cerebellar toxicity (Shields et al. 2012) or rhabdomyolysis (Bäckberg et al. 2015a; Lam et al. 2016). Severe adverse effects associated with inhalation of the designer drug methoxetamine include seizures, hyponatremia, and sinus bradycardia (Imbert et al. 2014). Regular ketamine use has been associated with potentially irreversible bladder dysfunction and subsequent renal impairment (Chu et al. 2007; Tsai et al. 2009). Animal studies suggest that this may also be a consequence of regular methoxetamine use (Dargan et al. 2014; Wang et al. 2017). In a survey of methoxetamine users, approximately one-fourth reported urinary symptoms (Lawn et al. 2016). The prevalence of urinary symptoms was related to the frequency of methoxetamine use during the previous month, but prior ketamine use could have also contributed to these symptoms (Lawn et al. 2016). Other dissociative designer drugs may also cause such severe urinary tract dysfunction, but detailed research has not been conducted. Additionally, the acute and chronic use of dissociative designer drugs potentially elicits wide-ranging effects on memory systems, similar to ketamine (Morgan and Curran 2006). Dissociative designer drugs have been involved in numerous fatal intoxications, mostly in combination with other designer drugs, including stimulants, opioids, cannabinoids, and psychedelics (Adamowicz and Zuba 2015; Bakota et al. 2016; Chiappini et al. 2015; De Jong et al. 2019; Elliott et al. 2015; Johansson et al. 2017; Krotulski et al. 2018; Kudo et al. 2015; Kusano et al. 2018; McIntyre et al. 2015c; Mitchell-Mata et al. 2017; Wiergowski et al. 2014; Wikström et al. 2013). Wallach and Brandt previously published a detailed overview of the clinical toxicology of individual PCP analogs (Wallach and Brandt 2018b) and diarylethylamine- and ketamine-based designer drugs (Wallach and Brandt 2018a).

## Synthetic cannabinoids

**Fig. 7:**
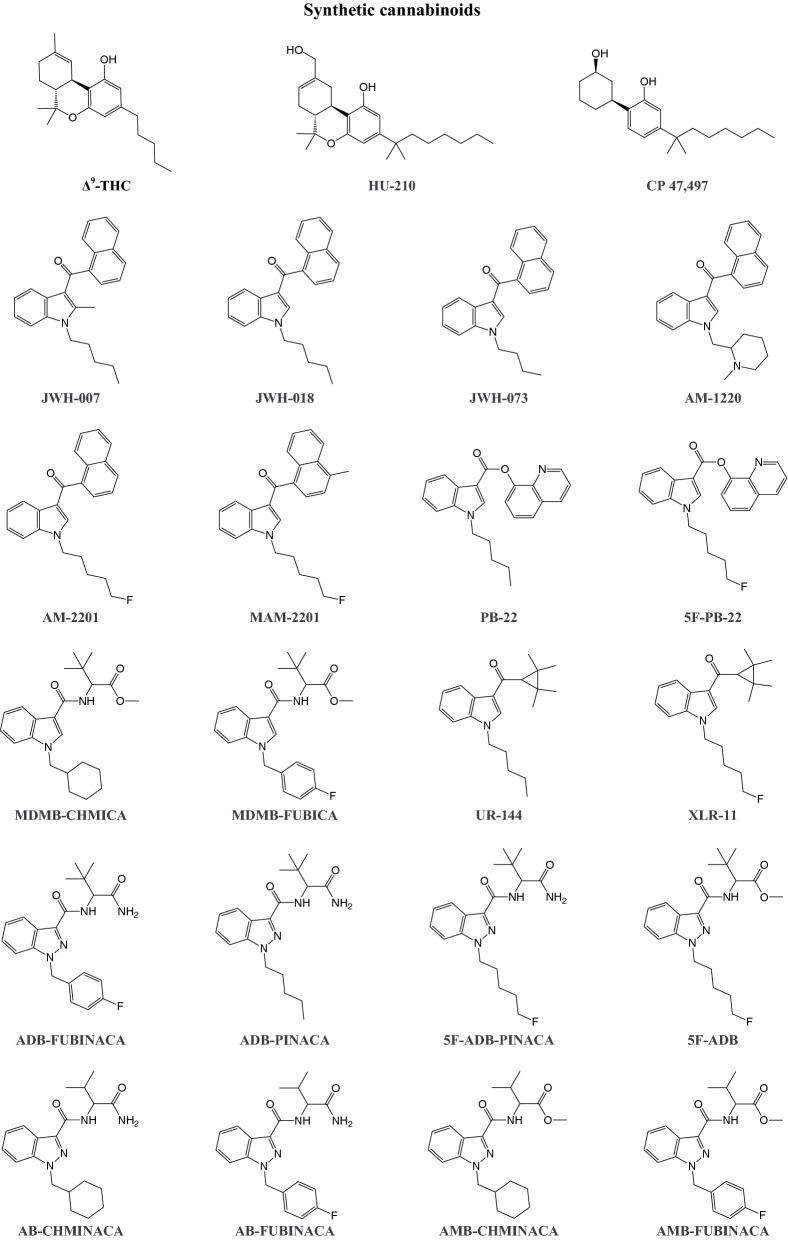
Structures of Δ^9^-THC and a selection of cannabinoid designer drugs. Full names of the substances are provided in the supplementary information

The endocannabinoid system is involved in various physiological functions, including cognition, behavior, memory, motor control, pain sensation, appetite, cardiovascular parameters, gastrointestinal motility, and immunoregulation (Le Boisselier et al. 2017). The term “cannabinoid” refers to a class of compounds that are produced by *Cannabis sativa* and *Cannabis indica*, and endogenous and exogenous ligands that interact with G protein-coupled cannabinoid type 1 (CB_1_) and CB_2_ receptors (Banister and Connor 2018; Le Boisselier et al. 2017). CB_1_ receptors are mainly expressed in the brain and modulate neurotransmitter signaling, whereas CB_2_ receptors are abundant in immune tissues (Banister and Connor 2018; Le Boisselier et al. 2017). The first synthetic cannabinoids were developed in the second half of the twentieth century to study human endocannabinoid receptor systems (Banister and Connor 2018; Le Boisselier et al. 2017; Trecki et al. 2015). Today, synthetic cannabinoids (Fig. 7) represent the largest and most structurally diverse class of designer drugs, and some of these compounds are similar to phyto- and endocannabinoids (Banister and Connor 2018; Trecki et al. 2015). Synthetic cannabinoids are often referred to as “Spice,” based on the first branded synthetic cannabinoid product. They are commonly applied to dried herbs that mimic cannabis (Banister and Connor 2018; Le Boisselier et al. 2017; Trecki et al. 2015). Desired effects of synthetic cannabinoids include relaxation, euphoria, and disinhibition, thus not significantly differing from desired effects of Δ^9^-tetrahydrocannabinol (Δ^9^-THC), the main psychoactive constituent of cannabis (Le Boisselier et al. 2017). However, compared with cannabis, synthetic cannabinoids have a less desirable effect profile and are associated with more severe adverse events that sometimes can result in death (Trecki et al. 2015; Winstock and Barratt 2013).

### Mechanism of action of synthetic cannabinoids

Various synthetic cannabinoids have been reported to bind to CB_1_ and CB_2_ receptors with higher efficacy at both receptors compared with Δ^9^-THC (Banister et al. 2015a, b, 2016, 2019; Gamage et al. 2018; Sachdev et al. 2019). Biased signaling at cannabinoid receptors or the disruption of mitochondrial homeostasis may play a role in the difference between clinical effects of Δ^9^-THC and synthetic cannabinoids, but research in this area is still in its infancy (Finlay et al. 2019; Silva et al. 2018, 2019). CB_1_ receptors are involved in multiple mechanisms that lead to the suppression of synaptic transmission. Compared with CB_2_ receptor expression, the predominance of CB_1_ receptors in the central nervous system indicates that they mainly mediate the psychoactive effects of synthetic cannabinoids (Atwood et al. 2010; Castillo et al. 2012; Kano et al. 2009; Le Boisselier et al. 2017). This assumption is strengthened by studies that reported that CB_1_ receptor antagonism but not CB_2_ receptor antagonism inhibits the synthetic cannabinoid-induced lowering of heart rate and body temperature in rodents (Banister et al. 2015a, 2019a). In vitro studies showed that various metabolites of synthetic cannabinoids retain some cannabimimetic activity, indicating that they could contribute to the pharmacological effects of the drugs (Longworth et al. 2017). In contrast, some substances that are promoted as cannabinoid designer drugs have only low in vitro affinity for cannabinoid receptors and fail to exert significant cannabinoid activity in vivo, thus calling into question their classification as synthetic cannabinoids (Banister et al. 2019b). Only a few synthetic cannabinoids have been studied to date with regard to their interactions with non-cannabinoid targets, with low or no affinity for most major neurotransmitter receptors (Wiley et al. 2016). This suggests that different effects of synthetic cannabinoids compared with Δ^9^-THC are mainly related to greater potency and efficacy at CB_1_ receptors, but possible effects on non-cannabinoid receptors and different signaling pathways that have not yet been discovered cannot be ruled out (Finlay et al. 2019; Wiley et al. 2016). Furthermore, pharmacokinetic differences may contribute to these differences.

### Adverse effects of synthetic cannabinoids

The most common adverse effects of synthetic cannabinoids include agitation, drowsiness, dizziness, confusion, hallucinations, hypertension, tachycardia, chest pain, nausea, and vomiting, which typically have a short duration and require only symptomatic or supportive treatment (Forrester 2012a; Forrester et al. 2011, 2012; Hoyte et al. 2012; Law et al. 2015; Tait et al. 2016). Nevertheless, compared with cannabis, complications that are associated with synthetic cannabinoid use are more frequent and in some cases, more severe (Alipour et al. 2019; Bäckberg et al. 2017; Mensen et al. 2019; Tait et al. 2016; Trecki et al. 2015). Various severe adverse events that are associated with synthetic cannabinoids have been reported. However, many of these cases were attributed to synthetic cannabinoid use based solely on statements by patients or witnesses, without analytical confirmation of the identity and amount of substances in bodily fluids or remaining drug products. Severe clinical complications that have been reported to be associated with synthetic cannabinoid use include convulsions and seizures (Adamowicz et al. 2017; Bäckberg et al. 2017; Bebarta et al. 2012; De Havenon et al. 2011; Gugelmann et al. 2014; Harris and Brown 2013; Hermanns-Clausen et al. 2013a, b; Hoyte et al. 2012; Lapoint et al. 2011; McQuade et al. 2013; Pant et al. 2012; Schep et al. 2015; Schneir and Baumbacher 2012; Tofighi and Lee 2012), status epileptics (Babi et al. 2017), catatonia (Khan et al. 2016; Leibu et al. 2013; Smith and Roberts 2014), delirium (Armenian et al. 2018a; Armstrong et al. 2019; Bäckberg et al. 2017; Schwartz et al. 2015; Tyndall et al. 2015), ischemic stroke (Bernson-Leung et al. 2014; Faroqui et al. 2018; Freeman et al. 2013; Moeller et al. 2017; Raheemullah and Laurence 2016; Takematsu et al. 2014; Wolff and Jouanjus 2017), intracranial hemorrhage (Aydin and Bakar 2019; Rose et al. 2015), pulmonary embolism (Raheemullah and Laurence 2016; Yirgin et al. 2018), pneumonia and pulmonary infiltrates (Alhadi et al. 2013; Alon and Saint-Fleur 2017; Berkowitz et al. 2015; Chinnadurai et al. 2016; Öcal et al. 2016), respiratory depression (Alon and Saint-Fleur 2017; Jinwala and Gupta 2012), supraventricular and ventricular arrhythmias (Davis and Boddington 2015; Ibrahim et al. 2014; Ozturk et al. 2019; Young et al. 2012), myocardial ischemia and infarction (Clark et al. 2015; Hamilton et al. 2017; Hirapara and Aggarwal 2015; McIlroy et al. 2016; McKeever et al. 2015; Mehta et al. 2017; Mills et al. 2018; Mir et al. 2011; Ozturk et al. 2019; Shah et al. 2016; Sherpa et al. 2015; Tse et al. 2014), takotsubo cardiomyopathy (Mohammed 2019), liver injury (Shahbaz et al. 2018), acute kidney injury (Argamany et al. 2016; Armstrong et al. 2019; Bhanushali et al. 2013; Buser et al. 2014; El Zahran et al. 2019; Gudsoorkar and Perez 2015; Kamel and Thajudeen 2015; Katz et al. 2016b; Kazory and Aiyer 2013; Srisung et al. 2015; Thornton et al. 2013; Zarifi and Vyas 2017; Zhao et al. 2015), hyperemesis syndrome (Argamany et al. 2016; Bick et al. 2014; Hopkins and Gilchrist 2013; Ukaigwe et al. 2014), and rhabdomyolysis (Adedinsewo et al. 2016; Argamany et al. 2016; Armstrong et al. 2019; Durand et al. 2015; El Zahran et al. 2019; Katz et al. 2016b; Sherpa et al. 2015; Sweeney et al. 2016; Zhao et al. 2015). Furthermore, various psychiatric adverse effects have been reported, including paranoia, psychosis, and ideations of self-harm and suicide (Akram et al. 2019; Altintas et al. 2016; Bassir Nia et al. 2019; Benford and Caplan 2011; Berry-Caban et al. 2013; Bonaccorso et al. 2018; Darke et al. 2019; Deng et al. 2018; Derungs et al. 2013; Durand et al. 2015; Every-Palmer 2010; Glue et al. 2013; Hermanns-Clausen et al. 2013b; Hobbs et al. 2018; Hurst et al. 2011; Kraemer et al. 2019; Martinotti et al. 2017; Meijer et al. 2014; Mensen et al. 2019; Müller et al. 2010; Oliveira et al. 2017; Oluwabusi et al. 2012; Papanti et al. 2013; Patton et al. 2013; Peglow et al. 2012; Roberto et al. 2016; Skryabin and Vinnikova 2019; Sweet et al. 2017; Thomas et al. 2012; Van Amsterdam et al. 2015; Van der Veer and Friday 2011; Welter et al. 2017; Yeruva et al. 2019). The sudden discontinuation of synthetic cannabinoid use in regular

## Psychedelics

**Fig. 8:**
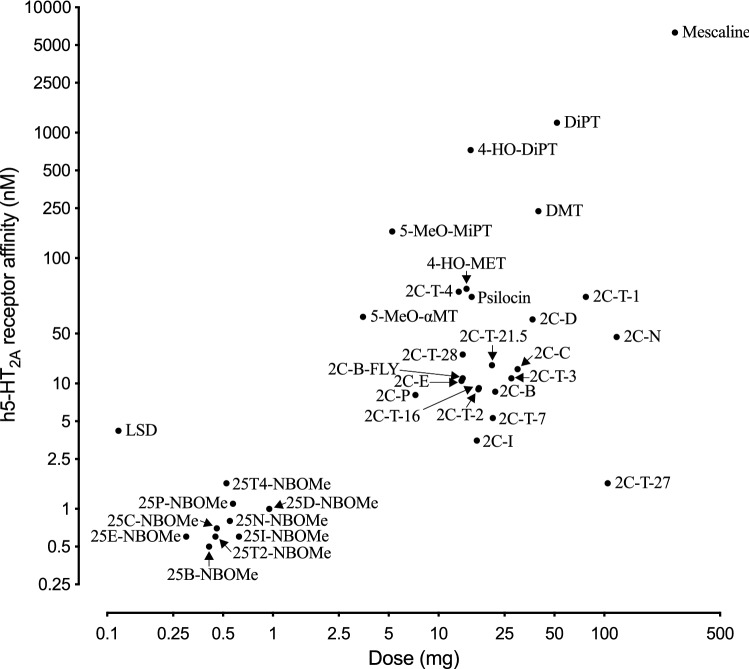
Correlation between reported clinical potencies and in vitro human 5-HT_2A_ receptor affinities of a variety of psychedelics. Figure modified from (Luethi and Liechti 2018). Full names of the substances and source of pharmacological data are provided in the supplementary information

Serotonergic psychedelics induce alterations of perception and cognitive states in users (Nichols 2004, 2016). Traditional psychedelics, such as the phenethylamine 3,4,5-trimethoxyphenethylamine (mescaline), the tryptamines *N,N*-dimethyltryptamine (DMT) and psilocybin, and the ergot alkaloid lysergic acid diethylamide (LSD), have a history of being used for religious purposes, as therapeutic agents, and as illicit black market drugs. Although psychedelics interact with various pharmacological targets, their psychedelic effects are mainly mediated by 5-HT_2A_ receptor agonism (Geyer and Vollenweider 2008; Kraehenmann et al. 2017; Madsen et al. 2019; Nichols 2004, 2016; Preller et al. 2018; Vollenweider et al. 1998). Affinity for 5-HT_2A_ and 5-HT_2C_ receptors is correlated with the amount of drug that induces psychedelic effects in humans (Fig. 8) (Luethi and Liechti 2018). Designer drug analogs of all the traditional psychedelic drugs are available, some of which were originally developed by industry or university laboratories but have eventually found their way onto the illicit drug market.

### Phenethylamines

**Fig. 9:**
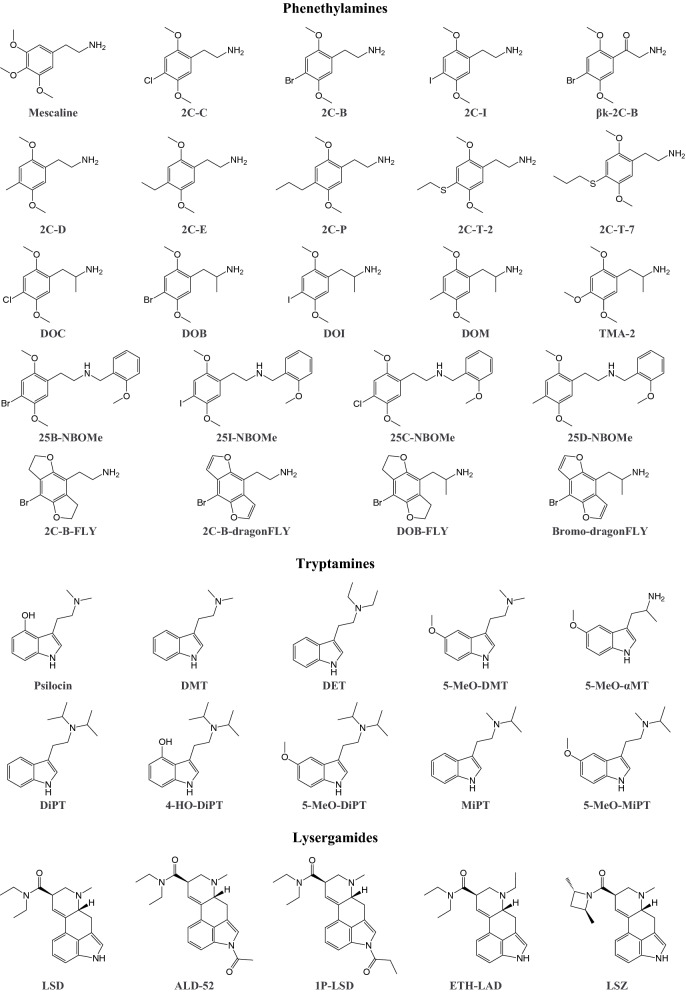
Examples of psychedelic phenethylamines, tryptamines, and lysergamides. Full names of the substances are provided in the supplementary information

Derivatives of mescaline comprise a large amount of psychedelic designer drugs (Fig. 9). The most widespread phenethylamine psychedelics are 2,5-dimethoxyphenethylamines, which bear a small lipophilic substituent at the 4-position (referred to as 2C series because they bear two carbon atoms between the benzene ring and amino group), and their slightly more potent α-methyl (amphetamine) analogs (Shulgin and Shulgin 1995). Psychedelic phenethylamine derivatives are mostly but not exclusively chemically modified at the phenyl ring. The introduction of an *N*-benzylmethoxy (“NBOMe”) group has been shown to increase the potency of the resulting derivatives (Eshleman et al. 2018; Halberstadt 2017; Heim 2004; Rickli et al. 2015c). The incorporation of 2′- and 5′-methoxy groups into rigid rings resulted in tetrahydrobenzodifuran and benzodifuran analogs that have been sold as designer drugs. These tetrahydrobenzodifuran and benzodifuran designer drugs are referred to as “FLY” and “dragonFLY” analogs, respectively, because of the shape of their chemical structure (Halberstadt et al. 2019; Trachsel et al. 2013). In one of the few clinical studies of a designer drug, 4-bromo-2,5-dimethoxyphenylethylamine (2C-B) was shown to induce euphoria, well-being, and changes in perception, and to have mild stimulant properties (González et al. 2015). 2C-B may thus be classified as a psychedelic with entactogenic properties, an effect profile that is similar to various other phenethylamine psychedelics (Shulgin and Shulgin 1995).

#### Mechanism of action of phenethylamines

Similar to other psychedelics, substituted phenethylamines mainly interact with serotonergic receptors, with the highest affinity for 5-HT_2A_ receptors (Eshleman et al. 2018; Kolaczynska et al. 2019; Luethi et al. 2018d; Rickli et al. 2015c). NBOMe derivatives have higher affinity for 5-HT_2A_ and 5-HT_2C_ receptors and lower affinity for 5-HT_1A_ receptors compared with their 2C analogs (Rickli et al. 2015c). At 5-HT_2A_ receptors, 2C and NBOMe derivatives were shown to be partial or full agonists, depending on the functional in vitro assay (Eshleman et al. 2014, 2018; Jensen et al. 2017; Kolaczynska et al. 2019; Luethi et al. 2018d, 2019c; Moya et al. 2007; Rickli et al. 2015c). NBOMe derivatives and most 2C derivatives have been shown to be partial agonists at 5-HT_2B_ receptors (Eshleman et al. 2018; Jensen et al. 2017; Kolaczynska et al. 2019; Luethi et al. 2018d; Rickli et al. 2015c). At 5-HT_2C_ receptors, 2C derivatives were shown to be partial or full agonists (Eshleman et al. 2014; Moya et al. 2007), and NBOMe derivatives were shown to be full agonists (Eshleman et al. 2018; Jensen et al. 2017). Consistent with the in vitro findings, psychedelic phenethylamines were shown to induce 5-HT_2A_-dependent behaviors in vivo, such as wet dog shakes, back muscle contractions, and a head twitch response (Elmore et al. 2018; Fantegrossi et al. 2005; Halberstadt et al. 2020; Halberstadt and Geyer 2014). In addition to interactions with serotonergic receptors, phenethylamine psychedelics have been shown to interact with other monoaminergic targets, including adrenergic, dopaminergic, and histaminergic receptors, monoamine transporters, and MAOs (Eshleman et al. 2018; Kolaczynska et al. 2019; Luethi et al. 2018d; Noble et al. 2018; Rickli et al. 2015c; Wagmann et al. 2019a). However, most of these interactions are weak compared with the potent interactions with serotonergic receptors. Therefore, they most likely have little or no pharmacological relevance to the actions of phenethylamine psychedelics. Rodent studies have suggested reinforcing effects for NBOMe derivatives that involve the dopaminergic system (Custodio et al. 2019; Seo et al. 2019). The NBOMe derivative 4-iodo-2,5-dimethoxy-*N*-(2-methoxybenzyl)phenethylamine (25I-NBOMe) was shown to increase extracellular dopamine, 5-HT, and glutamate levels in the rat frontal cortex (Herian et al. 2019), but unknown are the ways in which these findings translate to humans.

#### Adverse effects of phenethylamines

Most of the frequently reported adverse effects of phenethylamine psychedelics are shared by psychedelics of other chemical classes, including agitation, hallucinations, drowsiness, confusion, mydriasis, aggression, hyperthermia, hypertension, and tachycardia (Dean et al. 2013; Forrester 2013, 2014; Hermanns-Clausen et al. 2017; Hill et al. 2013; Iwersen-Bergmann et al. 2019; Rose et al. 2013; Srisuma et al. 2015; Stellpflug et al. 2014; Stoller et al. 2017; Tang et al. 2014; Topeff et al. 2011; Wood et al. 2015). Moreover, severe adverse effects have been linked to the use of psychedelic phenethylamines, including acute psychosis, seizures, coma, cerebral edema, long-lasting severe neurological impairment, serotonin syndrome, prolonged respiratory failure, renal failure, multi-organ failure, metabolic acidosis, and rhabdomyolysis (Bosak et al. 2013; Grautoff and Kähler 2014; Halberstadt 2017; Huang and Bai 2011; Miyajima et al. 2008; Srisuma et al. 2015; Tang et al. 2014; Wood et al. 2009). Furthermore, 1-(4-bromofuro[2,3-*f*][1]benzofuran-8-yl)propan-2-amine (Bromo-dragonFLY) has been associated with potent vasoconstriction, ischemia, and tissue necrosis in patients, which may be caused by the activation of serotonergic and adrenergic receptors combined with metabolic stability and long-lasting effects (Hill and Thomas 2011; Noble et al. 2018; Thorlacius et al. 2008; Wood et al. 2009). A remarkable case of mass intoxication with 4-ethyl-2,5-dimethoxyphenethylamine (2C-E) and Bromo-dragonFLY among 29 attendees of an esoteric weekend seminar was reported (Iwersen-Bergmann et al. 2019). Upon the arrival of paramedics, some of the seminar attendees were rolling on the ground and screaming, and others were unconscious or unresponsive. Several attendees exhibited severe delusions and physical symptoms, including generalized seizures, pain, respiratory distress, and tachycardia (Iwersen-Bergmann et al. 2019). In some severe cases, the use of psychedelic phenethylamines has even resulted in death (Curtis et al. 2003; Kueppers and Cooke 2015; Poklis et al. 2014; Shanks et al. 2015a; Topeff et al. 2011; Walterscheid et al. 2014). Adverse effects of different phenethylamine designer drugs are mostly comparable. However, a higher incidence of hallucinations, delusions, and single-episode seizures has been observed for NBOMe derivatives compared with 2C derivatives (Srisuma et al. 2015). This may be explained by the higher potency of NBOMe derivatives compared with most other phenethylamine psychedelics (Braden et al. 2006; Elmore et al. 2018; Halberstadt and Geyer 2014; Rickli et al. 2015c). Several reports have linked severe intoxication to substituted phenethylamines, but the lack of analytical confirmation of the drug prevents the direct attribution of adverse effects to phenethylamines. For example, a 43-year-old woman was reported to have developed severe headaches, progressive encephalopathy, and quadraparesis within 48 h after taking a liquid form of 2C-B that was synthesized according to a manual on the Internet (Ambrose et al. 2010). However, the patient’s urine tested positive for cannabinoids only, and a sample of the ingested drug could not be obtained for analysis (Ambrose et al. 2010). Similarly, a patient presented to an emergency department with hallucinations and agitation that progressed to status epilepticus after using of 4-chloro-2,5-dimethoxyamphetamine (DOC). In addition to the analytically confirmed presence of DOC, however, the toxicology screen was positive for cannabinoids and opioids, thus hampering the attribution of seizure development to DOC (Burish et al. 2015). Additionally, several fatalities from 2C derivative use have been reported in the media, but the accuracy of this information cannot be verified (Dean et al. 2013).

### Tryptamines

The core structure of tryptamine designer drugs contains an indole ring that is connected to an amino group by an ethyl side chain, a structural feature that is shared by 5-HT. DMT as an ingredient in the psychoactive brew ayahuasca and psilocybin that is contained in *Psilocybe* spp. mushrooms have been used in sociocultural and ritual contexts since ancient times. They have recently regained interest for their therapeutic use (Araújo et al. 2015; Carhart-Harris et al. 2018; Muttoni et al. 2019; Palhano-Fontes et al. 2019; Roseman et al. 2017). In addition to naturally occurring compounds, psychedelic properties of various synthetic tryptamines (Fig. 9) have been described (Shulgin and Shulgin 1997).

#### Mechanism of action of tryptamines

Similar to other psychedelics, 5-HT_2A_ receptor agonism plays a key role in mediating the psychedelic effects of naturally occurring and synthetic tryptamine psychedelics (Fantegrossi et al. 2008; Halberstadt 2016; Madsen et al. 2019; Vollenweider et al. 1998). Although mediating opposing functional effects on 5-HT_2A_ receptors, the concurrent activation of 5-HT_1A_ receptors has been suggested to contribute to the qualitative effects of tryptamine psychedelics, distinguishing them from phenethylamine psychedelics (Fantegrossi et al. 2008; Halberstadt and Geyer 2011; Nichols 2004, 2016; Winter et al. 2000). Most traditional and novel tryptamine psychedelics bind to 5-HT_1A_ and 5-HT_2A_ receptors with similar affinity. Some tryptamines are slightly more selective for one or the other receptor subtype (Rickli et al. 2016). However, various tryptamine psychedelics have been reported to be inactive at the 5-HT_1A_ receptor in functional assays at relevant concentrations (EC_50_ > 10 μM) or act as mostly partial agonists with significantly lower potency compared with 5-HT_2A_ receptors, at which tryptamine psychedelics act as moderate to full agonists (Blough et al. 2014; Rickli et al. 2016). At 5-HT_2B_ receptors, traditional and novel tryptamine psychedelics have very heterogeneous profiles, with different potencies and efficacies. For example, 5-methoxy-α-methyltryptamine (5-MeO-αMT) is a potent 5-HT_2B_ receptor full agonist with an EC_50_ in the low nanomolar range, whereas psilocin and 4-hydroxy-*N*-methyl-*N*-ethyltryptamine (4-HO-MET) are inactive at the 5-HT_2B_ receptor (EC_50_ > 20 μM) (Rickli et al. 2016). Tryptamine designer drugs have been shown to bind to 5-HT_2C_ receptors but with slightly lower affinity compared with 5-HT_2A_ receptors (Rickli et al. 2016). In addition to their primary effects at serotonergic receptors, tryptamines have been shown to bind to various targets in vitro, including adrenergic, dopaminergic, and histaminergic receptors (Klein et al. 2018; Rickli et al. 2016). Furthermore, unlike phenethylamine or lysergamide psychedelics, many tryptamine psychedelics interact with monoamine transporters at pharmacologically relevant concentrations. In addition to some interactions with the DAT and NET for some compounds, tryptamines have the most potent transporter interactions at the SERT (Blough et al. 2014; Cozzi et al. 2009; Rickli et al. 2016). DMT and other tryptamine psychedelics have been reported to elicit 5-HT efflux, suggesting that they are transporter substrates (Blough et al. 2014; Cozzi et al. 2009; Rickli et al. 2016). In contrast, other tryptamine psychedelics, including psilocin, act as transporter inhibitors that are devoid of substrate activity (Rickli et al. 2016). In addition to interactions with transmembrane monoamine transporters, substrate activity at the VMAT has been described for tryptamine psychedelics (Cozzi et al. 2009). Tryptamines are prone to metabolism by MAOs, and MAO inhibitors counteract extensive degradation of tryptamines after oral use (Halberstadt et al. 2012; Ott 1999, 2001; Riba et al. 2015).

#### Adverse effects of tryptamines

Similar to other psychedelics, tryptamine psychedelics alter perception and can induce psychological disturbances in users, including acute psychosis (Meatherall and Sharma 2003; Nichols 2004, 2016; Shulgin and Shulgin 1997; Taljemark and Johansson 2012). Adverse effects of tryptamine designer drugs include restlessness, disorientation, clouding of consciousness, confusion, hallucinations, amnesia, catalepsy, mydriasis, tachypnea, hypertension, and tachycardia (Alatrash et al. 2006; Itokawa et al. 2007; Jovel et al. 2014; Muller 2004; Smolinske et al. 2005). 5-Methoxy-*N*,*N*-diisopropyltryptamine (5-MeO-DiPT) use has been associated with hallucinogen-persisting perception disorder and was proposed to play a role in the development of prolonged delusions (Fuse-Nagase and Nishikawa 2013; Ikeda et al. 2005). In severe cases, the use of tryptamine designer drugs has resulted in acute renal failure and rhabdomyolysis (Alatrash et al. 2006; Jovel et al. 2014). Furthermore, several fatalities after the use of tryptamine designer drugs have been reported (Boland et al. 2005; Sklerov et al. 2005; Tanaka et al. 2006).

### Lysergamides

Several derivatives of LSD have been described in the scientific literature, and such derivatives are increasingly emerging as designer drugs (Fig. 9) (Brandt et al. 2016, 2017a, b, 2018, 2019; Shulgin and Shulgin 1997; Troxler and Hofmann 1957). The LSD-derived designer drugs 1-acetyl-LSD (ALD-52), 1-propionyl-LSD (1P-LSD), and 1-butyryl-LSD (1B-LSD) have been shown to be metabolized to LSD in vitro and are thus considered precursors of LSD with very similar effects (Wagmann et al. 2019b). Whereas self-reported effects of some LSD analogs are similar to LSD but with slightly weaker or less pleasurable effects, other LSD analogs have been reported to be distinctively less potent or significantly differ from LSD in terms of effects (Coney et al. 2017; Shulgin and Shulgin 1997).

#### Mechanism of action of lysergamides

Several LSD-derived designer drugs induce a head twitch response in mice, and pretreatment with a selective 5-HT_2A_ receptor antagonist abolished the 1P-LSD-induced head twitch response (Brandt et al. 2017a; Brandt et al. 2016). This indicates that, similar to LSD, 5-HT_2A_ receptor activation mediates the behavioral effects of LSD analogs (Kraehenmann et al. 2017; Liechti 2017; Preller et al. 2017, 2018). Additionally, 5-HT_1A_ receptor activation likely contributes to the qualitative effects of lysergamide designer drugs similarly to LSD and tryptamine psychedelics (Fantegrossi et al. 2008; Halberstadt and Geyer 2011; Nichols 2004, 2016; Rickli et al. 2016; Winter et al. 2000). In addition to differences in affinity, LSD-derived designer drugs may activate 5-HT_2A_ receptors with lower relative potency compared with LSD, but more research is needed to test this hypothesis (Brandt et al. 2017a). Furthermore, unclear are the ways in which the behavioral effects of lysergamide designer drugs in animals translate to humans.

#### Adverse effects of lysergamides

Little is known about the adverse effects of lysergamide designer drugs. One case of a 17-year-old male who developed anxiety, hallucinations, restlessness, elevations of blood pressure, palpitations, and tachycardia after ingesting 1P-LSD was reported (Grumann et al. 2019). 1P-LSD was confirmed as an ingredient of the ingested blotter paper but could not be detected in urine or serum samples, despite being sufficiently stable in these matrices. However, LSD was detected in both samples, thus strengthening the assumption that 1P-LSD is readily metabolized to LSD in humans (Grumann et al. 2019). The patient reported that he recently used the stimulant phenmetrazine derivative 3-FPM. The low serum concentrations of 3-FPM that were detected at the time of hospital admission are, however, not expected to result in acute effects (Grumann et al. 2019). The symptoms of this 1P-LSD intoxication case are consistent with reported adverse effects of LSD, which is known to potentially cause psychological disturbances and moderately increase body temperature, blood pressure, and heart rate (Dolder et al. 2016; Schmid et al. 2015). Acute physiological adverse effects of LSD include difficulty concentrating, imbalance, feelings of exhaustion, dizziness, headache, dry mouth, lack of appetite, and nausea (Dolder et al. 2016; Schmid et al. 2015). Nichols and Grob recently reviewed the risk of LSD toxicity in users, which they concluded was very low (Nichols and Grob 2018). The few cases of fatality that were attributed to LSD toxicity were either associated with massive overdoses or physical restraint, or they were potentially caused by drugs that remained undetected in the toxicological analysis (Nichols and Grob 2018). Currently, no evidence suggests that any of the currently available lysergamide designer drugs are significantly more toxic than LSD.

## Performance-enhancing designer drugs

Designer doping agents have become increasingly popular outside of the professional athletic community and include anabolic steroids, peptide hormones, growth factor mimetics, and hormone and metabolic modulators (Joseph and Parr 2015; Poplawska and Blazewicz 2019; Rahnema et al. 2015; Weber et al. 2017). Such substances are mainly used for performance and image enhancement, exerting effects through several different mechanisms within the hormone system (Graham et al. 2009; Kicman 2008). Adverse effects that are associated with performance-enhancing designer drugs include secondary hypogonadism, gynecomastia, infertility, hypertension, ischemic stroke, cardiotoxicity, hepatotoxicity, and renal failure (Rahnema et al. 2015). In addition to substances that are taken to enhance athletic performance and appearance, designer drugs that are taken to enhance sexual performance, such as phosphodiesterase-5 inhibitors with no known safety profile, have also appeared. These substances may potentially induce visual disturbances or severe drug–drug interactions (Venhuis et al. 2008).

## Miscellaneous risks associated with designer drug use

In addition to adverse effects that are associated with specific classes of designer drugs, some general risks are essentially the same as for traditional drugs of abuse. For example, quality assurance is not guaranteed for clandestine designer drugs. A lack of information about purity, mislabeling, pharmaceutical impurities, and hazardous cutting agents can pose a risk for drug users. A series of patients who presented to a hospital with coagulopathy and bleeding diathesis that were related to long-acting anticoagulant rodenticide adulterants of synthetic cannabinoids exemplifies their potentially fatal consequences (Devgun et al. 2019; Kelkar et al. 2018). Potentially severe drug–drug interactions are a risk when more than one substance is used, including prescription medications (Contrucci et al. 2020; Inan et al. 2020). Byproducts and impurities can pose such risks as septum perforation when insufflated or necrotic ulcers and infections when injected (Lafferty et al. 2016; Parks et al. 2015). Hallucinogen-persisting perception disorder has been associated with psychedelics, cannabinoids, and psychostimulants, manifesting in prolonged or reoccurring perceptual symptoms (Ikeda et al. 2005; Orsolini et al. 2017; Skryabin et al. 2019). The neurological and psychological changes that are associated with designer drugs can impair safe driving, and driving under the influence can severely jeopardize traffic safety (Adamowicz and Lechowicz 2015; Maas et al. 2015; Musshoff et al. 2014).

## Concluding remarks

Designer drugs are often used in combination with other substances, thus hindering precise evaluations of the degree of involvement of individual substances to clinical toxicity in patients. Furthermore, designer drugs may remain undetected by routine drug screenings. Nevertheless, the pharmacological and toxicological profiles of most designer drug classes are similar to their related traditional drugs of abuse. Stimulants primarily act as substrates or inhibitors of monoamine transporters. Intoxication with stimulants mostly manifests as sympathomimetic adverse effects, the treatment of which is mainly supportive. Benzodiazepines may be given to control agitation, hypertension, and convulsions. Certain stimulants, including MDMA, have a marked serotonergic profile. Their associated adverse effects, such as serotonin syndrome, can be potentially severe clinical complications. Agonism at μ-opioid receptors mediates the main pharmacological effects of opioids, and GABA_A_ and GABA_B_ receptors drive the effects of designer benzodiazepines and GHB analogs, respectively. Sedatives, including synthetic opioids and GHB analogs, pose a risk of cardiorespiratory arrest, especially when they are used in combination with other depressants, such as alcohol and benzodiazepines. Initial patient care should focus on protecting the airways and maintaining breathing and circulation. Naloxone is an effective antidote to opioid toxicity. Dissociative designer drugs act as NMDA receptor antagonists and induce adverse effects that are similar to the dissociative anesthetics ketamine and PCP. Compared with Δ^9^-THC, synthetic cannabinoids act as more potent and effective agonists of CB_1_ and CB_2_ receptors, but the predominance of CB_1_ receptors in the central nervous system suggests that they mainly mediate the psychoactive effects. Compared with cannabis, effects of synthetic cannabinoids are less desirable, and adverse effects are more severe. Serotonergic psychedelics alter perception and cognition mainly through 5-HT_2A_ receptor agonism. In addition to psychological disturbances, psychedelics may induce physical adverse effects, which are usually short-lived. Rarely, designer drug use can lead to severe psychiatric and physical complications and even death. Single-drug use and more precise knowledge of substance identity, potency, and purity can reduce the risks of designer drug use.

## Electronic supplementary material

Below is the link to the electronic supplementary material.

Supplementary file1 (XLSX 38 kb)
